# Exploration of Dihydrothieno[2,3-*c*] Isoquinolines As Luminescent Materials and Corrosion Inhibitors

**DOI:** 10.1021/acsomega.2c03361

**Published:** 2022-10-19

**Authors:** Islam S. Marae, Mahmoud H. Mahross, Badriah S. Al-Farhan, Mohamed Abdel-Hakim, Etify A. Bakhite, Marwa M. Sayed

**Affiliations:** † Chemistry Department, Faculty of Science, Assiut University, Assiut 71516, Egypt; ‡ Chemistry Department, Faculty of Science, Al-Azhar University, Assiut 71524, Egypt; § Chemistry Department, Faculty of Girls for Science, King Khalid University, Abha 62529, Saudi Arabia; ∥ Chemistry Department, Faculty of Science, New Valley University, El-Kharja 72511, Egypt

## Abstract

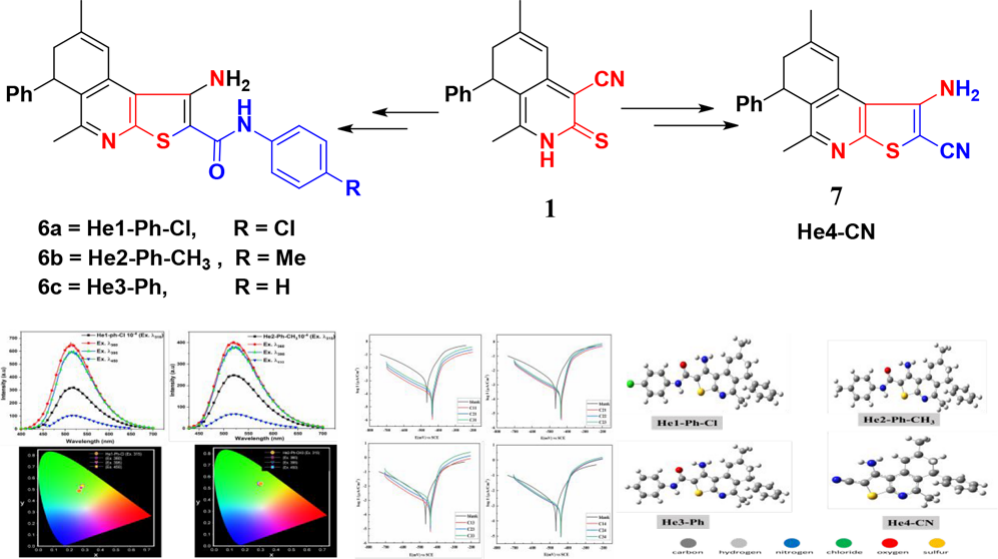

The reaction of the starting compound, 7-acetyl-4-cyano-1,6-dimethyl-8-phenyl-7,8-dihydroisoquinoline-3(2*H*)-thione, with some *N*-aryl-2-chloroacetamides or chloroacetonitrile, in the presence of sodium acetate trihydrate, gave the corresponding substituted 3-methylsulfanyl-7-acetyl-4-cyano-1,6-dimethyl-8-phenyl-7,8-dihydroisoquinolines. Upon heating of the latter compounds with sodium methoxide in methanol, they underwent intramolecular Thorpe-Zeigler cyclization, affording the target isomers 1-amino-2-(substituted)-5,8-dimethyl-6-phenyl-6,7-dihydrothieno[2,3*-c*]isoquinolines (DHTIQs). The chemical structures of all produced substances were characterized by elemental and spectral analyses. The photophysical characteristics of the produced DHTIIQs (He1-Ph-Cl, He2-Ph-CH_3_, He3-Ph, and He4-CN) have been investigated as luminous compounds. Potentiodynamic, surface morphology, and theoretical calculations were used to study the behavior of the synthesized DHTIQs as corrosion inhibitors on mild steel in a 1.0 M sulfuric acid solution.

## 1 Introduction

Luminescent compounds have received significant interest in material science due to their specific applications for fluorescent sensors, light sources, optical-recording systems, and information displays.^1−7^ Recently, we published a paper on luminescent compounds containing a thieno[2,3-*b*]pyridine moiety, which showed aggregation-induced emission (AIE) behavior with high absolute quantum yields.^8^ On the other hand, organic corrosion inhibitors that contain heteroatoms such as nitrogen, oxygen, sulfur, and phosphorus work well in a wide variety of acidic solutions.^9,10^ The efficiency of the inhibitor depends on its stability, and the inhibitor particles must contain molecules or atoms capable of electrostatic attraction with the metal surface through the transfer of electrons.^11^ Only a few studies have been published on the use of quinoline, isoquinoline, and some of its derivatives as corrosion inhibitors in various mediums.^12−16^ Most previous studies indicate that isoquinoline derivatives are effective inhibitors and their inhibition efficiencies increase as their concentrations increase.^17−19^ Moreover, the literature survey revealed only a few publications on the synthesis and applications of 7,8-dihydroisoquinoline derivatives. Therefore, the present study focuses on synthesizing and characterizing some 6,7-dihydrothieno[2,3-*c*]isoquinoline derivatives and studying their applications as fluorescent materials and corrosion inhibitors.

## 2 Results and Discussion

### 2.1 Synthesis of Dihydrothienoisoquinoline (DHTIQ) Derivatives

**Scheme 1:**
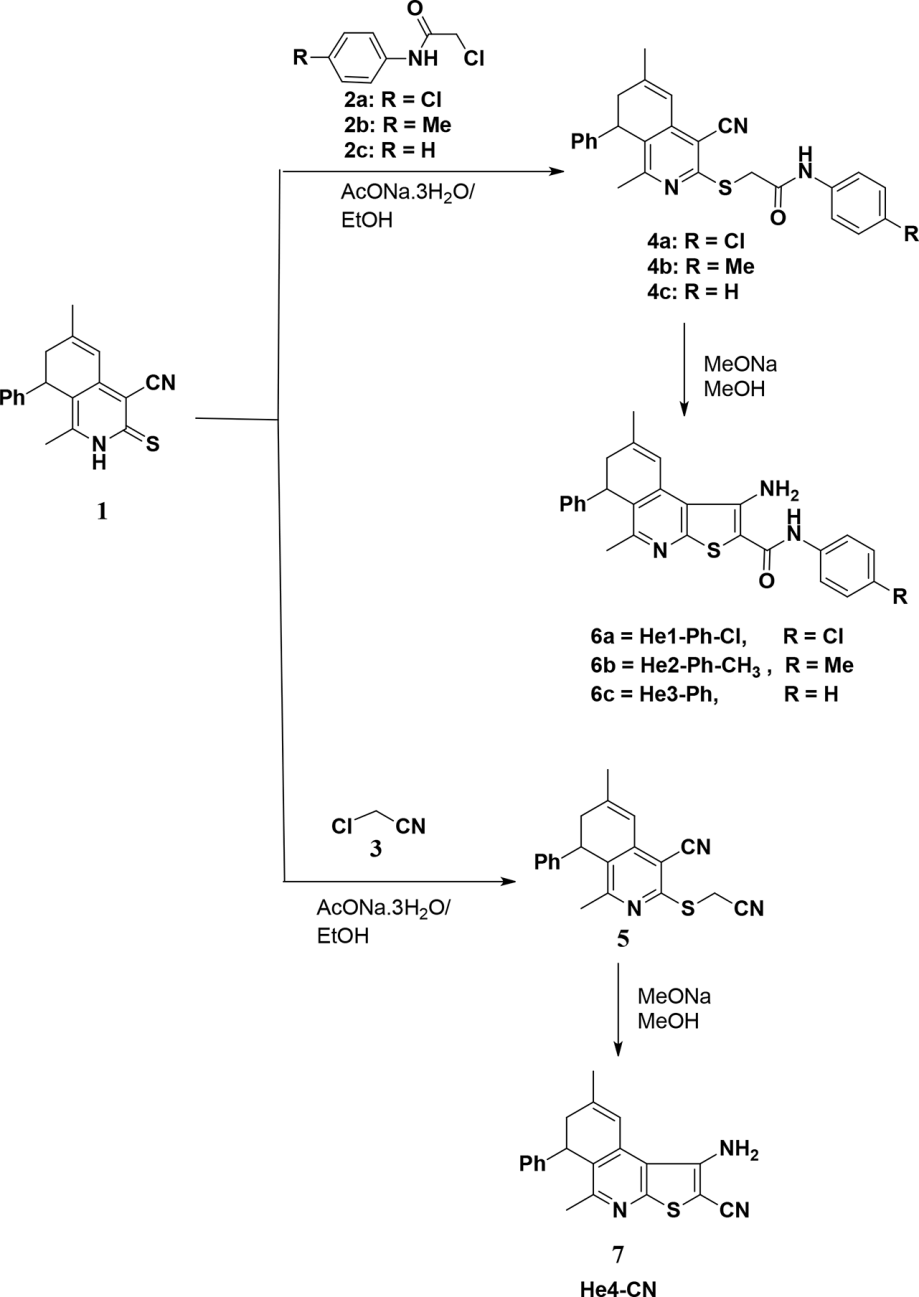
Synthesis of Dihydrothienoisoquinoline Derivatives (DHTIQs; **6a**–**6c** and **7**)

The target dihydrothienoisoquinoline products were prepared by starting from a readily available compound, 7-acetyl-4-cyano-1,6-dimethyl-8-phenyl-7,8-dihydroiso-quinoine-3-(2*H*)-thione (**1**). Thus, the reaction of compound **1** with *N*-aryl-2-chloroacetamide **2a**–**2c** or chloroacetonitrile (**3**) by refluxing in ethanol in the presence of sodium acetate trihydrate gave the corresponding 2-(substituted methyl sulfanyl)-7,8-dihydroisoquinoline-4-carbonitriles **4a**–**4c** or **5**, respectively (Scheme 1). The subsequent compounds (**4a**–**4c** or **5**) underwent intramolecular Thorpe-Zeigler cyclization after being treated with sodium methoxide in methanol, yielding the appropriate isomers. 1-Amino-2-(substituted)-6,7-dihydrothieno[2,3-*c*]isoquinolines **6a**–**6c** or **7** (DHTIQs) were coded as **6a**, He1-Ph-Cl; **6b**, He2-Ph-CH_3_; **6c**, He3-Ph; and **7**, He4-CN (Scheme 1).

### 2.2 Characterization of Dihydrothienoisoquinoline (DHTIQ) Derivatives

Elemental and spectral analyses confirmed the chemical structures of all newly synthesized compounds (*cf*. Experimental Section and Figures S1–S9).

### 2.3 Photophysical Properties

**Figure 1:**
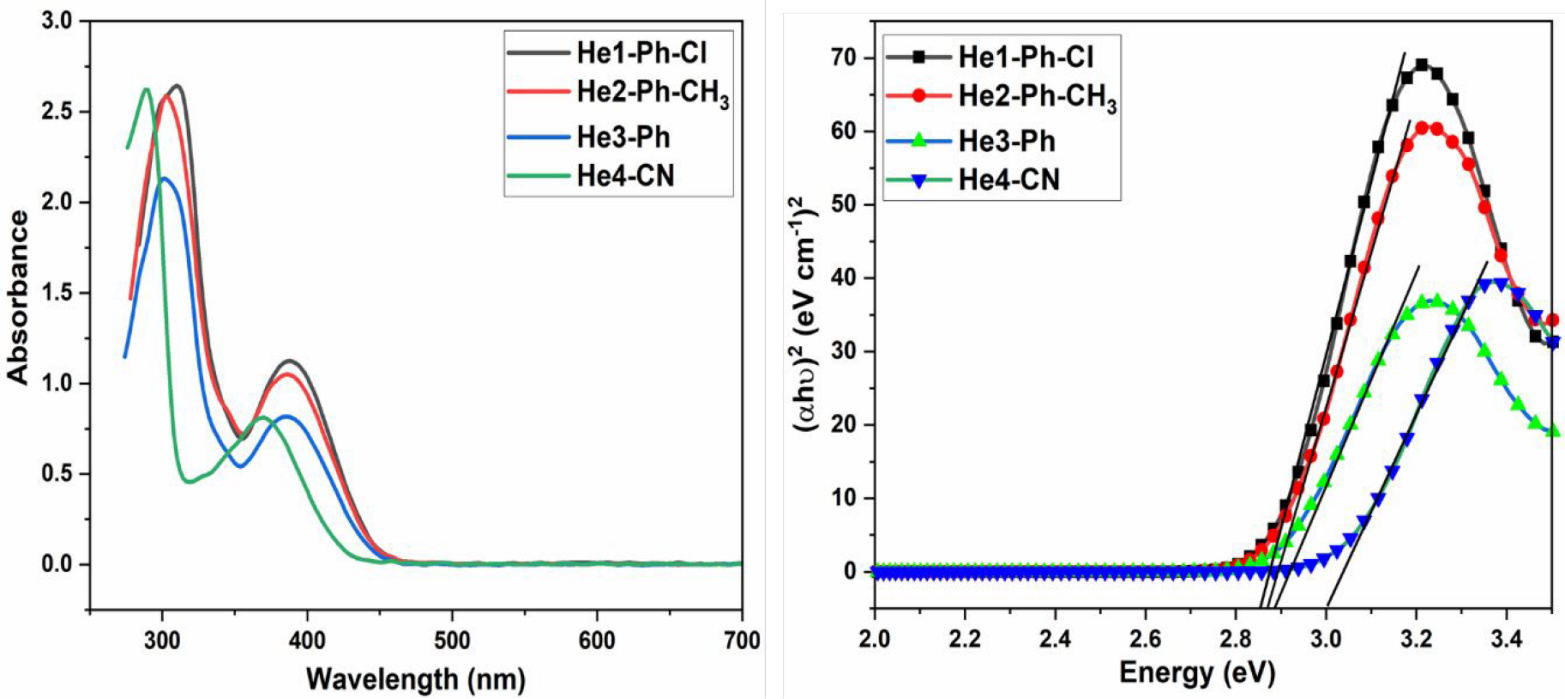
UV–visible spectra (left) and energy band gaps (right) of He1-Ph-Cl (**6a**), He2-Ph-CH_3_ (**6b**), He3-Ph (**6c**), and He4-CN (**7**).

Figure 1 shows the measured compounds’ UV–visible and energy band gap spectra. The optical properties are measured in DMF solution at a concentration of 10^–4^ M. Each of these compounds exhibits two absorption bands, at λ_max_= 310 nm and λ_max_= 388 nm for He1-Ph-Cl (**6a**), at λ_max_= 302 nm and λ_max_= 386 nm for He2-Ph-CH_3_ (**6b**), at λ_max_= 298 nm and λ_max_= 386 nm for He3-Ph (**6c**), and at λ_max_= 290 nm and λ_max_= 368 nm for He4-CN (**7**). These bands are attributed to n−π*and π–π* electronic transitions. The N-arylcarbamoyl group at position 2 of the DHTIQs increases conjugation, resulting in a higher λ_max_ π–π* transition maximum and absorbance intensity. The replacement in the *N-*phenylcarbamoyl moiety also caused the red shift and change in absorbance intensity. The change is greater for the electron-withdrawing group (e.g., chlorine atom) than for the electron-donating one (e.g., a methyl group). It tends to lower the electrons’ density, leading to a decrease in HOMO level.^20^ On the other hand, the absence of a(n) (un)substituted *N*-phenylcarbamoyl group from the structure produces a blue shift, and the existence of an electron-withdrawing cyano group lowers the conjugation λ_max_ of n−π*and π–π* transitions by reducing the HOMO level and hence increasing excitation energy.^21,22^ The values of the energy band gap of DHTIQs (**6a** = 2.85 eV, **6b** = 2.87 eV, **6c** = 3.00 eV, and **7** = 2.88 eV) are inconsistent with their structures. With no substituent on the phenyl ring of the N-arylcarbamoyl group, the amount of energy required for excitation is reduced. In addition, the presence of electron-withdrawing groups in that ring facilitates the transition and requires little energy.

**Figure 2:**
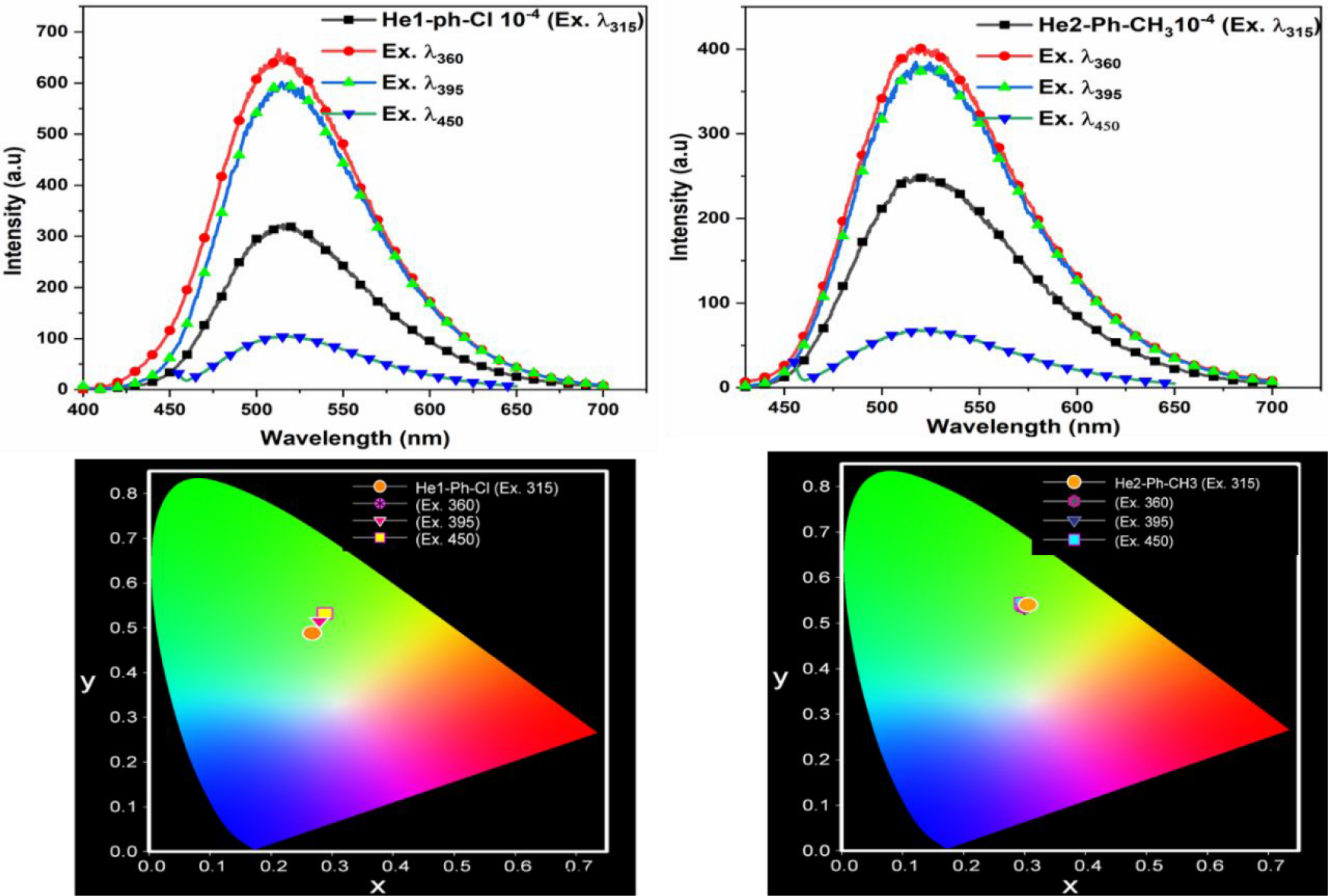
Emission spectra of He1-Ph-Cl (**6a**) and He2-Ph-CH_3_ (**6b**) at various excitation wavelengths and their CIE chromaticity diagrams.

**Figure 3:**
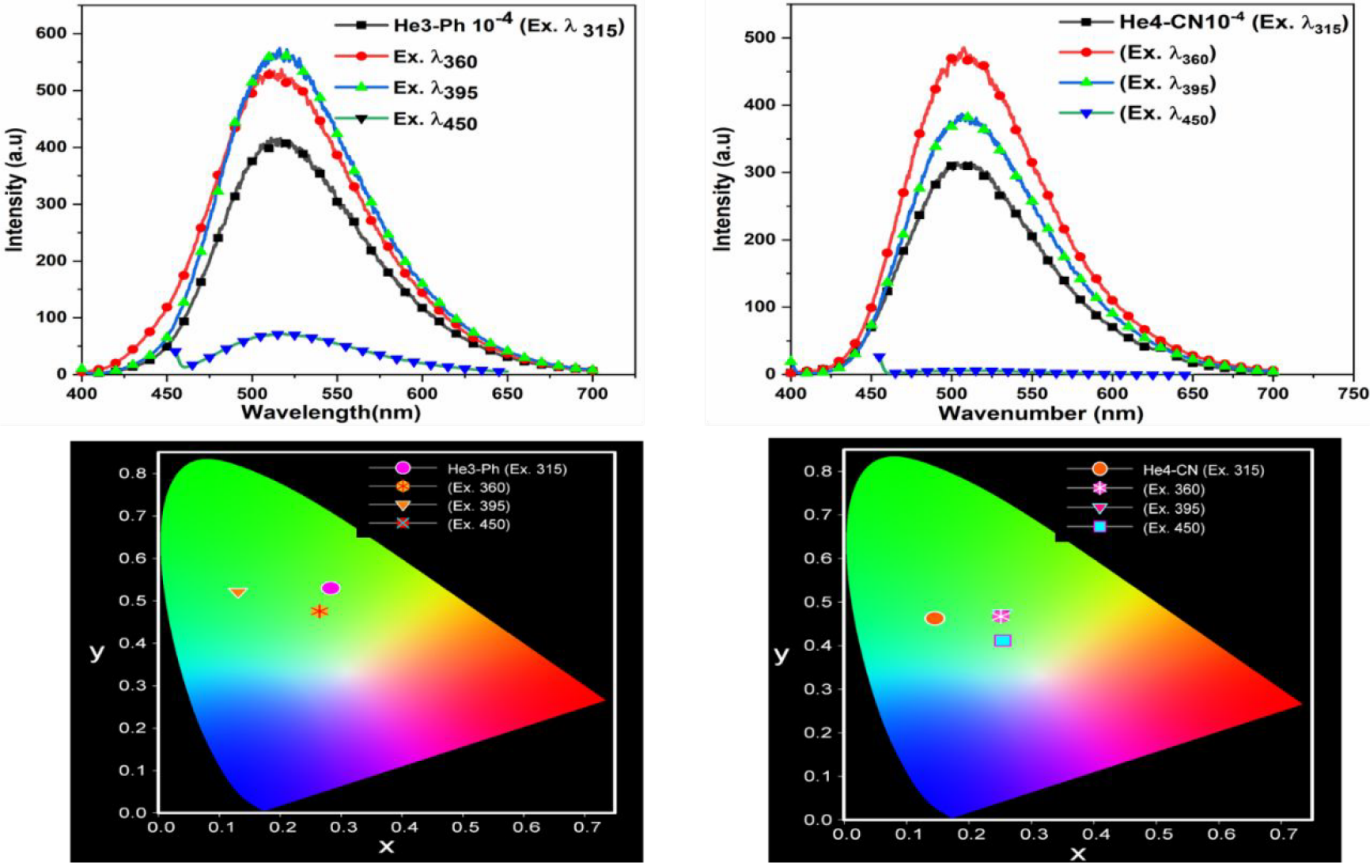
Emission spectra of He3-Ph (**6c**) and He4-CN (**7**) at various excitation wavelengths and their CIE chromaticity diagrams.

**Figure 4:**
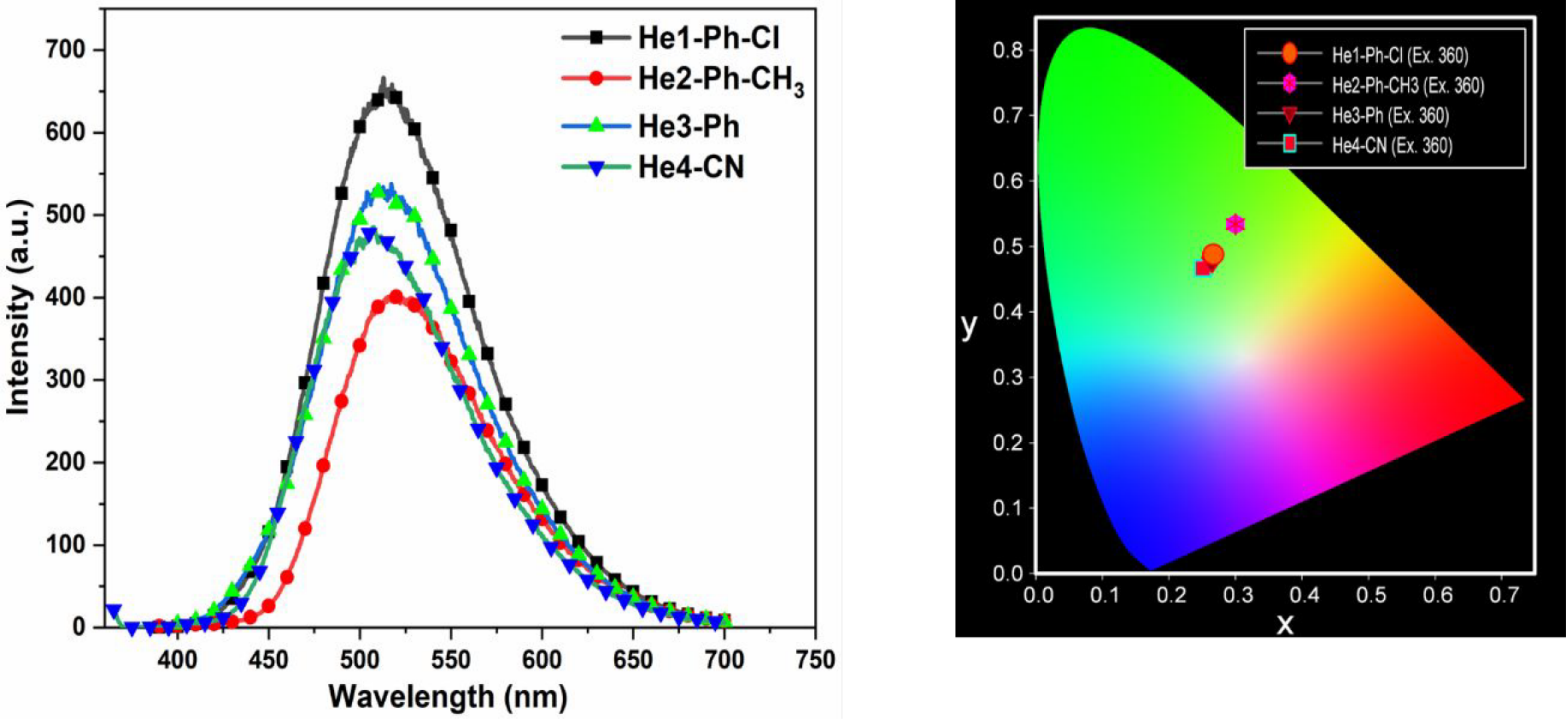
Emission spectra (left) and CIE chromaticity diagram (right) of compounds He1-Ph-Cl (**6a**), He2-Ph-CH_3_ (**6b**), He3-Ph (**6c**), and He4-CN (**7**) at λ_ex_ = 360 nm.

The emission spectra of these DHTIQs at different excitation wavelengths with a concentration of 10^–4^ in DMF are shown in Figures 2 and 3. Upon excitation of the samples with various wavelengths (λ_ex_ = 315 nm, λ_ex_ = 360 nm, λ_ex_ = 395 nm, λ_ex_ = 450 nm), the results indicate that (i) all DHTIQs exhibit nearly the same emission wavelength (at λ_em_ = 514 nm for He1-Ph-Cl, at λ_em_ = 521 nm for He2-Ph-CH_3_, at λ_em_ = 513 nm for He3-Ph, and at λ_em_ = 509 nm for He4-CN) with different intensities and, (ii) for all of them, the highest emission intensity is found at λ_ex_ = 360 nm except for He3-Ph, which has its highest emission intensity at λ_ex_ = 395 nm. The emission behavior of the DHTIQs at λ_ex_ = 360 nm is displayed in Figure 4; among all compounds, the highest emission intensity is observed for He1-Ph-Cl. The emission intensities exhibited by DHTIQs can be arranged in the following order: He1-Ph-Cl > He3-Ph > He4-CN > He2-Ph-CH_3_; this may be attributed to the structural effect on emission spectra via stabilization of the π–π* transition and destabilization of the n−π* one, i.e., the resonance effect caused by changing the substituent in the phenyl ring of the *N*-arylcarbamoyl group at position 2.^23,24^ The color of emission is quantized by the Commission Internationale de l’Eclairage (CIE) chromaticity diagram (Figures 2 and 3). The CIE coordinates are given in Table S1. All samples emit a green color with different excitation wavelengths, except for He2-Ph-CH_3_, which emits a yellowish-green color, confirming the dependence of fluorescence color on the structure of the compound.

### 2.4 Electrochemical Studies

#### 2.4.1 Open Circuit Potential OCP

Figure S10 displays the curves between *E* (mV) vs time (min) at a current of zero for MS immersed in the blank solution and three concentrations (100, 200, and 500 ppm) of the tested inhibitors (He1-Ph-Cl, He2-Ph-CH_3_, He3-Ph, and He4-CN). *E*_ss_ moves to the negative potential for blank solution curves relative to *E*_im_. This change is due to the breakdown of the oxide film on the MS surface until it reaches the corrosion cell’s *E*_ss_. The addition of various concentrations of the testing inhibitors caused the *E*_ss_ value to shift to greater positive potential than the blank solution. The latter effect is due to the formation of an adsorbed layer of inhibitor molecules on the active sites of the MS surface. Data extracted from OCP are illustrated in Table S2; it is clear that the increasing concentration of tested inhibitors from 100 to 500 ppm causes *E*_ss_ to shift to a positive direction more than for the blank solution.

#### 2.4.2 Tafel Polarization

**Figure 5:**
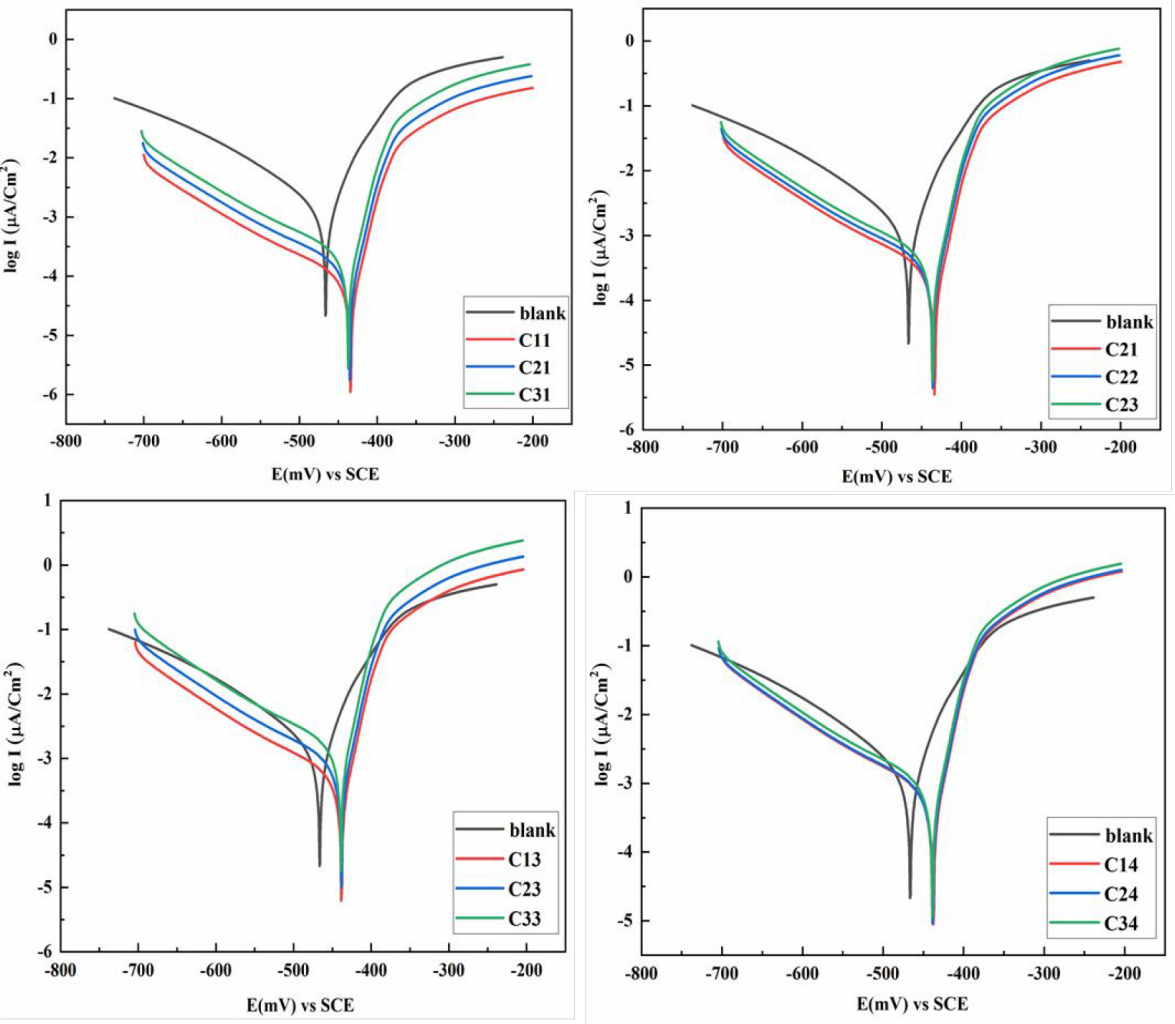
Potentiodynamic polarization curves for the corrosion of mild steel in 1.0 M H_2_SO_4_ solution before and after adding concentrations of (C11–C31) He1-Ph-Cl, (C21–C23) He2-Ph-CH_3_, (C13–C33) He4-CN, and (C14–C34) He3-Ph, respectively.

Figure 5 shows potentiodynamic polarization curves of the corrosion of mild steel in 1.0 M H_2_SO_4_ solution before and after adding various concentrations of the tested inhibitors. It observed that the presence of DHTIIQs in different concentrations causes shifting in Tafel slopes. This indicated that (i) the adsorption of inhibitor molecules on the surface of MS electrodes and (ii) the *E*_corr_ of used inhibitors differs positively from that of the blank solution, and the difference does not reach 85 mV, which proved that these inhibitors are mixed ones^25^ where there are reductions in the anodic and cathodic Tafel slope. Table 1 records the parameters extracted from TF such as *I*_corr_, *E*_corr_, CR, IE%, and θ of MS with and without inhibitors. In the absence of studied inhibitors, it is clear that *I*_corr_ increases to reach 2990 (μA/cm^2^) and CR increases to reach 2757 mpy. In addition to different concentrations of inhibitors of the blank solution, decreases in each *I*_corr_, CR, and IE% were observed. For example, *I*_corr_, CR, and IE% for MS exposed to 500 ppm of the He1-Ph-Cl derivative is 150 μA/cm^2^, 138 mpy, and 95%, respectively. For MS exposed to 500 ppm of the He1-Ph-Cl derivative, it is observed decreasing in *I*_corr_, CR, and IE%.

**Table 1:** Potentiodynamic Polarization Parameters of Mild Steel Immersed in 1.0 M H_2_SO_4_ with Different Types of DHTIQs

inhibitors (ppm)		*I* (μA/cm^2^)	CR	IE%	θ
1.0 M H_2_SO_4_		2990	2757		
He1-Ph-Cl	100	420	387	86.0	0.86
	200	255	235	91.5	0.91
	500	150	138	95.0	0.95
He2-Ph-CH_3_	100	600	553	79.9	0.80
	200	490	452	83.6	0.84
	500	365	337	87.8	0.88
He4-CN	100	690	636	76.9	0.77
	200	620	572	79.3	0.79
	500	498	459	83.3	0.83
He3-Ph	100	777	716	74.0	0.74
	200	655	604	78.1	0.78
	500	566	522	81.1	0.81

#### 2.4.3 Adsorption Isotherm

**Figure 6:**
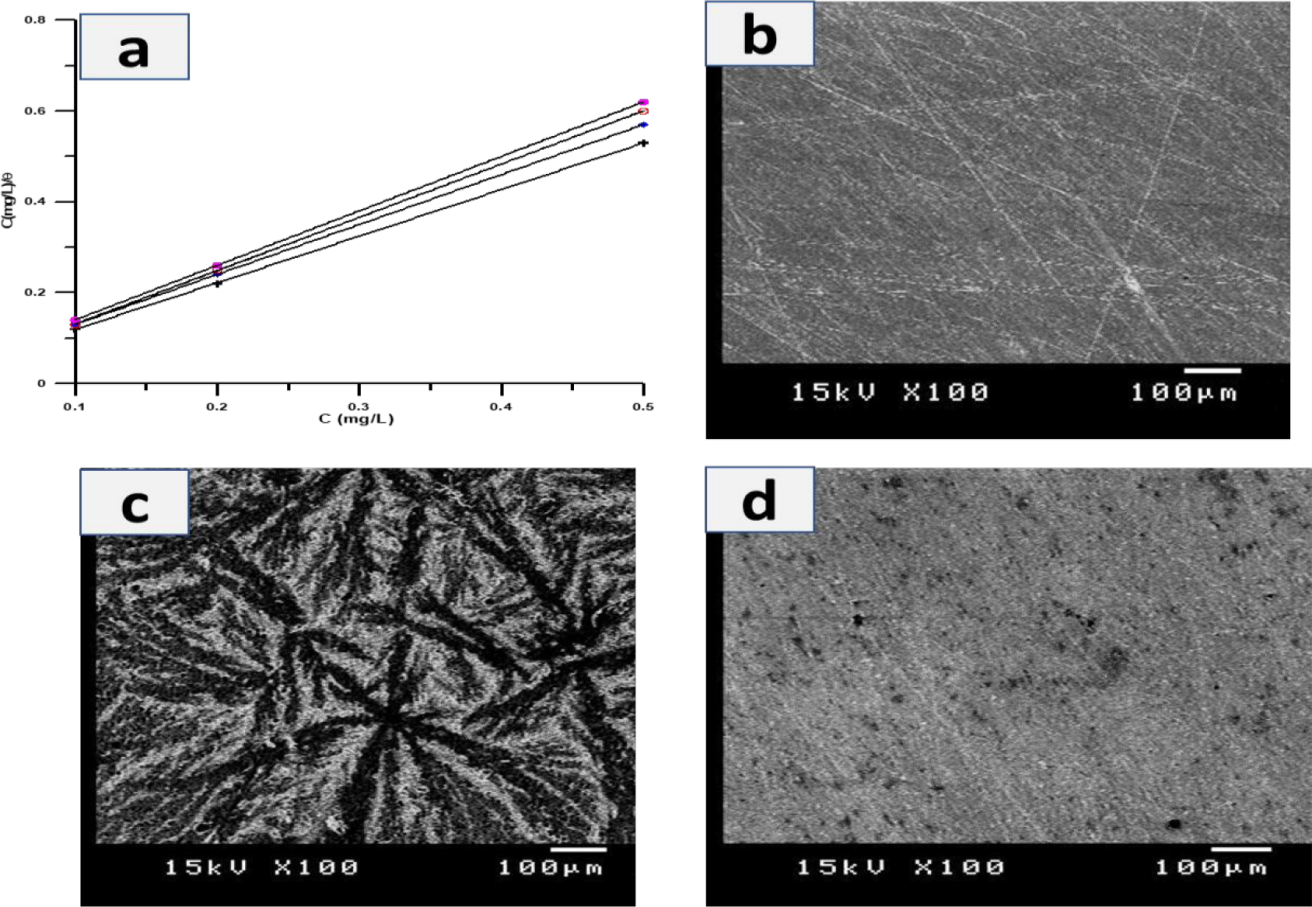
(a) Langmuir adsorption isotherm plots for the corrosion of mild steel in 1 M H_2_SO_4_ solution after adding concentrations of He1-Ph-Cl, He2-Ph-CH_3_, He4-CN, and He3-Ph, respectively. (b) SEM of bar mild steel electrode, (c) polished mild steel electrode in 1.0 M H_2_SO_4_, and (d) mild steel immersed in a blank solution with 500 ppm of He1-Ph-Cl.

The adsorption isotherm is carried out by covering the inhibitor molecules on MS surfaces (θ = IE%/100) in corrosive media. The nature of the interaction between the MS surface and the inhibitor molecules solution is determined by the sort of adsorption that occurs (physisorption or chemisorption). The Langmuir isotherm is shown by plotting *C*_inh_ against *C*_inh_/θ as follows in eq 1, illustrated in Figure 6a; *K*_ads_ obtained from this plotting is equal to the inverse of the intercept and slope near the unity value. By following the values of the slope and *K*_ads_, it is clear that the type of this adsorption is a Langmuir adsorption isotherm.
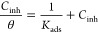
1The equilibrium constant of adsorption (*K*_ads_) is related to the Gibbs free energy of adsorption (Δ*G*_ads_) by the following modified eq 2:
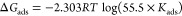
2Table S3 records the values of Δ*G*_ads_, slope, log *K*_ads_ ,and correlation coefficient *R*^2^ for MS exposed to free solution before and after adding different inhibitors. It is observed that the negative values of Δ*G*_ads_ are indicated spontaneously.^26^ In this work, the importance of Δ*G*_ads_ varied from −20.6 to −19.6 kJ/mol; these values emphasized that the adsorption process between the MS surface and inhibitor molecules obeyed physisorption.

#### 2.4.4 SEM Examination

SEM gives information about the shape of the surface morphology alteration according to acid attack of the surface of MS electrodes before and after adding the highest concentration of the best inhibitor, 500 ppm of He1-Ph-Cl, with high inhibition efficiency. Figure 6b illustrates SEM pictures of the MS surface after eliminating the corrosion product from the surface (after polishing). Figure 6c shows images of the MS surface after being immersed for 24 h in a blank solution; it is observed from these pictures that the surface of MS had a strong attack and deterioration because of the reaction of the ions of aggressive media sulfate anions.^27^Figure 6d shows a picture of an MS surface immersed in 500 ppm of He1-ph-Cl for 24 h in the presence of a blank solution; it is obvious that the acid media attack on the MS surface was detected or delayed, resulting in the formation of a protective layer of inhibitor molecules on the surface of MS electrodes, which is the cause for MS corrosion protection.

#### 2.4.5 Quantum Calculation

**Figure 7:**
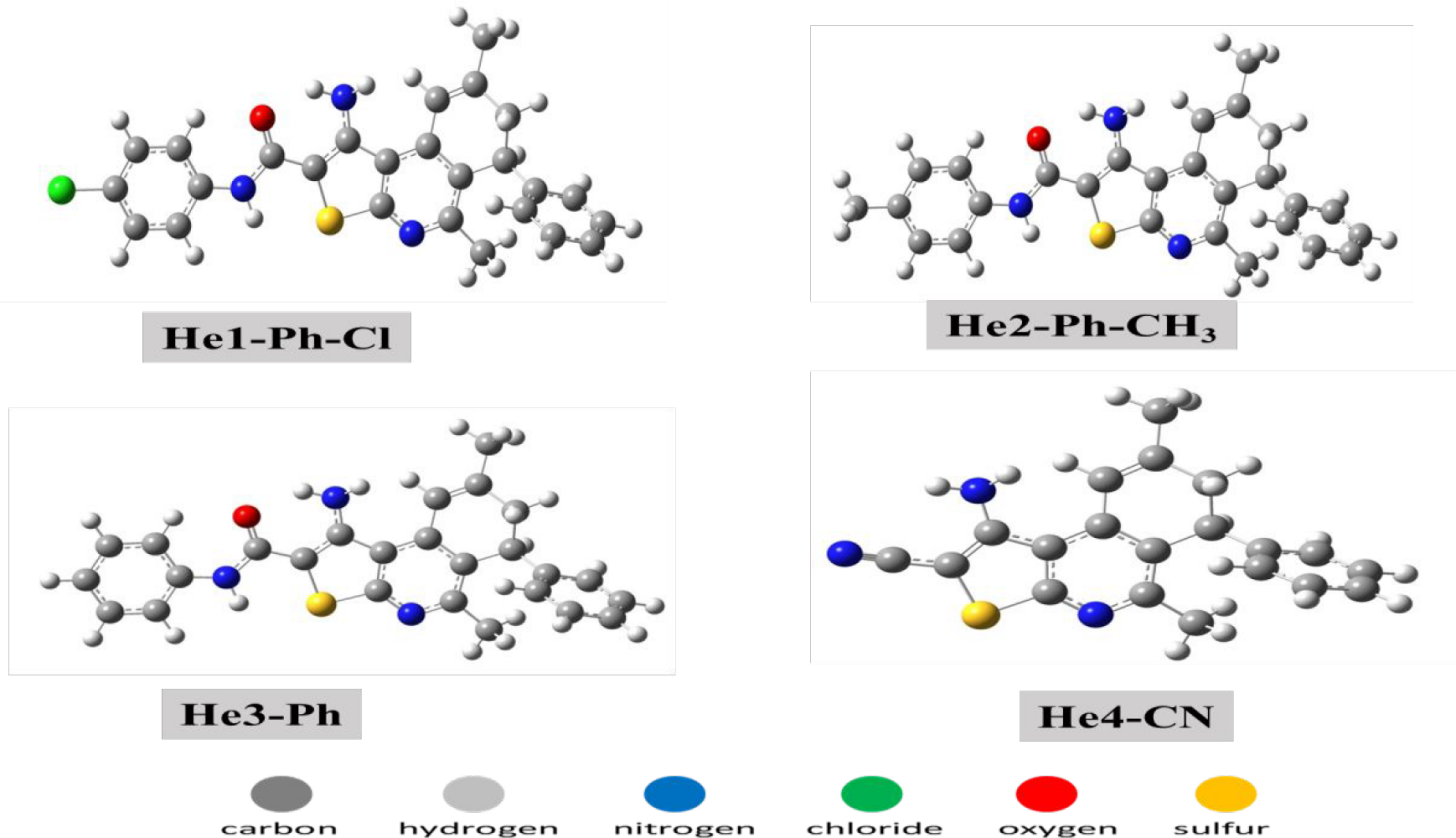
Optimized structures of DHTIQs by method and base set DFT/B3LYP/6-311G(d,p).

**Figure 8:**
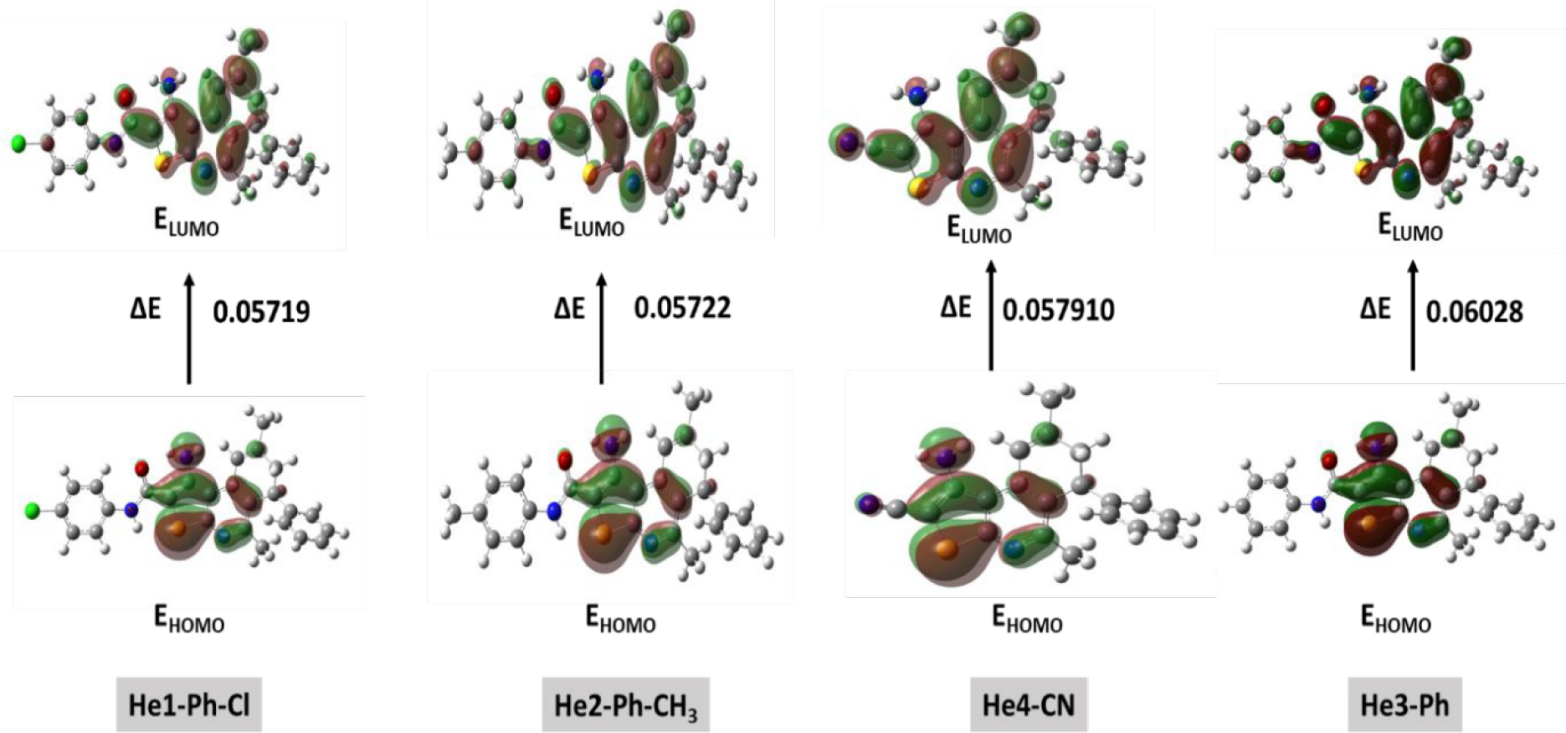
Molecular orbitals of DHTIQs by method and base set DFT/B3LYP/6-311G (d,p).

**Figure 9:**
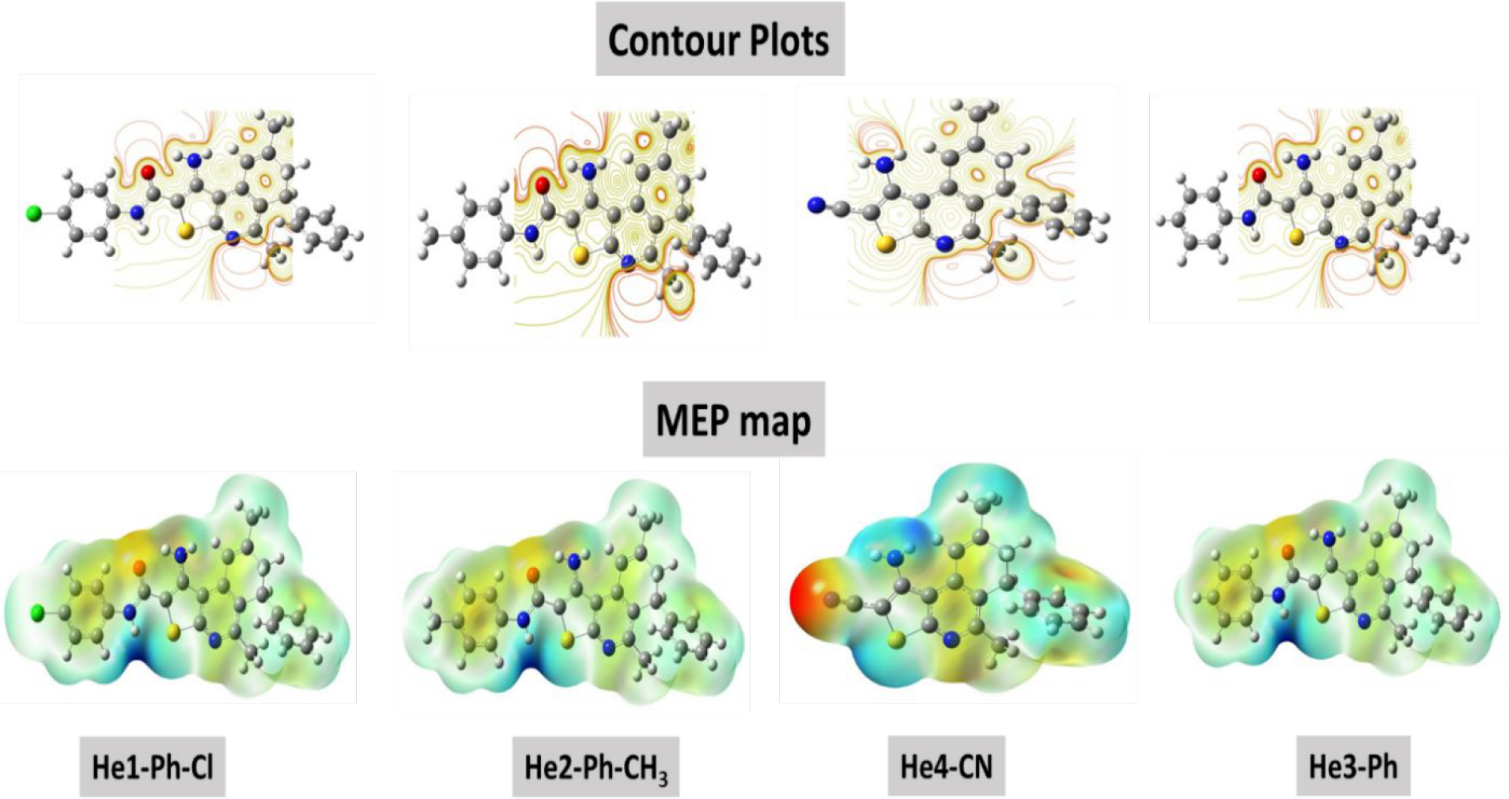
Contour plots and MEB maps of DHTIQs by method and base set DFT/B3LYP/6-311G (d,p).

Quantum calculations introduce information on some descriptors that describe the stability or activity of inhibitor derivatives that can correlate with experimental inhibition efficiency. *E*_HOMO_ means the molecule’s ability to eject electrons that are equal in mean ionization potential by inverse charge of the value from these descriptors. In the first step, good corrosion inhibitors are those organic molecules that present electrons into the unoccupied orbital of the metal and accept free electrons from the metal.^28,29^ Likewise, lower values of EG, which are calculated from *E*_HOMO_ + *E*_LUMO_ orbitals, lead to good inhibition efficiency because the energy releasing the electron from the highest occupied orbital will be small.^30^ In this study, comparing the stable and active inhibitors, it is clear from Table 2 that the EG value of He1-Ph-Cl is lower than those of other inhibitors that have the highest value, which means that He1-Ph-Cl is more active than other derivatives (more stable). Because each electronegativity χ and chemical potential μ have been linked to *E*_HOMO_ and *E*_LUMO_, He1-Ph-Cl has a high value for each and a lower value for EG. Also, because hardness η is connected to EG, He1-Ph-Cl, which has a low EG, must have a lower hardness η than other compounds with a high EG, causing the other compounds to have a higher hardness value. Figure 7 shows the optimized compounds of the tested inhibitors under the obvious mentioned method and base set. Figure 8 depicts *E*_HOMO_ and *E*_LUMO_ for all tested inhibitors; it is clear from these images that *E*_HOMO_ sites on the structure differ from *E*_LUMO._ The sites of *E*_HOMO_ and *E*_LUMO_ for all tested compounds differ from one another, indicating the previously mentioned differences in activity or stability. Figure S11 shows the Mulliken charge population analysis (MCPA) for all tested inhibitors, which represents the more or less negative charges, which emphasized which inhibitor molecules having highly negative charges have been used to find out the adsorption centers of the inhibitors.^31^ The higher negative charge of the adsorbed center makes it easier for an atom to donate its electrons to the vacant 3d orbital of the tested metal.^32,33^ There are alternative methods used to predict the active sites in the tested molecules known as molecular electrostatic potential (MEP) maps and contour plots. By different colors of MEP, maps can be used to determine the negative and positive charges on the molecule. The electro-love area is represented as the red and yellow area on the MEP map; on the other hand, the nucleus-love site has the light blue and dark blue locations on the MEP map. Additionally, in the MEP profiles, there are two colors, which are yellow and red, and these colored lines are related to the positively charged and negatively charged regions, respectively. Figure 9 shows MEP maps and contour plots of the investigating inhibitors; the electro-love area is mainly observed around the N, S, and O atoms in the center of the molecule and contour plots.

**Table 2:** Some Descriptors of Quantum Studies of DHTIQs by Method and Base Set DFT/B3LYP/6-311G (d,p)

comp.	Δ*E*	*I*	*u*	η	σ	*S*	–Δ*N*
He1-Ph-Cl	0.05719	0.2730	0.2444	0.0286	34.9725	0.0143	8.5669
He2-Ph-CH_3_	0.05722	0.2733	0.2447	0.0286	34.9545	0.0143	8.5546
He4-CN	0.05910	0.2744	0.2449	0.0296	33.8407	0.0148	8.2860
He3-Ph	0.06028	0.2774	0.2472	0.0301	33.1802	0.0151	8.2036

## 3 Experimental Section

### 3.1 Reagents and Solvents

All chemicals and solvents used herein are purchased from Sigma-Aldrich at analytical grade and are used as received without purification. Analytical TLC was performed using 2.5 × 5 cm aluminum plates coated with a 0.25 mm thickness of silica gel (60F-254). The visualization was accomplished with iodine and under a UV lamp.

### 3.2 Instrumentations

All compounds’ melting points were measured using Gallan-Kamp equipment and are uncorrected. TLC was used to assess the purity of the compounds. A Shimadzu 470 IR-spectrophotometer was used to record the infrared (IR) spectra (KBr). ^1^H NMR spectra were recorded on a 500 MHz spectrometer using CDCl_3_ or DMSO-*d*_6_ as a solvent and tetramethylsilane (TMS) as an internal reference (chemical shifts were given in ppm (δ) and coupling constants (*J* values) in Hertz (Hz)). The splitting patterns were designated as singlet (s), doublet (d), doublet of doublets (dd), triplet (t), quartet (q), or multiplet (m). The spectra of UV–vis were measured using a Shimadzu mini1240. The fluorescence emission spectra are performed at room temperature using a Hitachi F-7100 FL Spectrophotometer. The Gaussian 09 program is used for quantum chemical calculations^34^ via the density functional theory method with the B3LYP functional in a gaseous phase^35^ and the 6-311++G (d,p) basis set. To conduct open circuit potential linear and Tafel plot polarization tests in electrochemical studies, the 352/252 model corrosion measuring technique is utilized in conjunction with an EG&G potentiostat/galvanostat, model 273A, that operates on IBM software. Scanning electron microscope (SEM) analysis was performed at the unit of electronic microscopy (JEOL, JSM5400LV) at Assuit University.

### 3.3 Synthesis of the New Target Dihydrothienoisoquinoline Derivatives (DHTIQs)

#### 3.3.1 Synthesis of 7-Acetyl-4-cyano-1,6-dimethyl-8-phenyl-7,8-dihydroisoquinoline-3(2*H*)-thione (**1**)

It was prepared according to our previous method.^36^

#### 3.3.2 Synthesis of Substituted 3-Methylsulfanyl-7-acetyl-4-cyano-1,6-dimethyl-8-phenyl-7,8-dihydroisoquinolines **4a**–**4c** and **5**: General Method

A mixture of compound **1** (2.92 g, 0.01 mol), appropriate *N*-aryl-2-chloroacetamide **2a**–**2c** or chloroacetonitrile (**3**; 0.01 mol), and sodium acetate trihydrate (1.50 g, 0.011 mol) in ethanol (60 mL) was heated under reflux for 1 h and then left at room temperature overnight. The formed solid was collected and recrystallized from ethanol to give colorless crystals of **4a**–**4c** or **5**.

##### 3.3.2.1 3-[*N*-(4-Chlorophenyl)carbamoylmethylsulfanyl]-4-cyano-1,6-dimethyl-8-phenyl-7,8-dihydroisoquinoline (**4a**)

**4a** was synthesized by using *N*-(4-chlorophenyl-2-chloroacetamide (**2a**). m.p.: 168–169 °C. Yield: 4.9 g (90%). IR: 3294–2964 (NH); 3112, 3067 (C–H, arom.); 2922 (C–H, aliph.); 2217 (C≡N); 1680 (C=O); 1645 (C=N). ^1^H NMR (CDCl_3_): 9.54 (s, 1H, NH); 7.34–7.36 (d, *J* = 10, 2H, ArH); 7.20–7.25 (m, 5H, ArH); 6.98–7.00 (d, *J* = 10, 2H, ArH); 6.64 (s, 1H, C^5^H); 4.21–4.23 (d, *J* = 10, IH, C^8^H); 3.86–4.00 (dd, 2H, SCH_2_); 2.98–3.01 (dd, 1H, C^7^H); 2.53–2.56 (d, 1H, C^7^H); 2.41 (s, 3H, CH_3_); 1.90 (s, 3H, CH_3_). ^13^C NMR (126 MHz, DMSO-*d*_6_): δ 166.86, 159.63, 158.73, 149.33, 145.50, 142.94, 138.47, 129.16, 129.12, 127.51, 127.47, 127.20, 125.02, 121.21, 118.11, 115.51, 99.41, 37.90, 37.28, 35.38, 24.59, 22.47. C_26_H_22_ClN_3_OS (459.12): C, 67.89; H, 4.82; N, 9.13%. Found: C, 68.09; H, 4.70; N, 9.01%.

##### 3.3.2.2 4-Cyano-1,6-dimethyl–8-phenyl-3-[*N*-(4-tolyl)carbamoylmethylsulfanyl]-7,8-dihydroisoquinoline (**4b**)

**4b** was synthesized by using *N*-(4-tolyl)-2-chloroacetamide (**2b**). m.p.: 162–163 °C. Yield: g (90%). IR: 3294–3264 (NH); 3112 (C–H, arom.); 2920 (C–H, aliph.); 2207 (C≡N); 1678 (C=O). ^1^H NMR (CDCl_3_): 9.36 (s, 1H, NH); 7.23–7.29 (m, 5H, ArH); 7.05–7.07 (d, 2H, *J* = 10, ArH); 6.98–7.00 (d, *J* = 10, 2H, ArH); 6.63 (s, 1H, C^5^H); 4.20–4.22 (d, *J* = 10, IH, C^8^H); 3.86–4.01 (dd, 2H, SCH_2_); 2.97–3.01 (dd, 1H, C^7^H); 2.52–2.56 (d, *J* = 16, 1H, C^7^H); 2.41 (s, 3H, CH_3_); 2.27 (s, 3H, CH_3_); 1.90 (s, 3H, CH_3_). ^13^C NMR (126 MHz, DMSO-*d*_6_): δ 166.42, 159.66, 158.87, 149.31, 145.46, 142.95, 137.08, 132.73, 129.60, 129.12, 127.52, 127.20, 124.95, 119.67, 118.10, 115.56, 99.34, 37.90, 37.25, 35.33, 24.61, 22.50, 20.95. MS: *m*/*z* 439, M, +, 2%. Anal. calcd for C_27_H_25_N_3_OS (439.17): C, 73.77; H, 5.73; N, 9.56%. Found: C, 73.52; H, 5.66; N, 9.71%.

##### 3.3.2.3 4-Cyano-1,6-dimethyl-8-phenyl-3-phenylcarbamoylmethylsulfanyl-7,8-dihydro-isoquinoline (**4c**)

**4c** was synthesized by using *N*-phenyl-2-chloroacetamide (**2c**). m.p.: 159–160 °C. Yield: 4.3 g (84%). IR: 3297–3267 (NH); 3060 (C–H, arom.); 2923 (C–H, aliph.); 2220 (C≡N); 1679 (C=O); 1645 (C=N). ^1^H NMR (CDCl_3_): 9.47 (s, 1H, NH); 6.99–7.41 (m, 10H, ArH); 6.64 (s, 1H, C^5^H); 4.21–4.23 (d, *J* = 10, IH, C^8^H); 3.88–4.02 (dd, 2H, SCH_2_); 2.96–3.01 (dd, 1H, C^7^H); 2.52–2.56 (d, 1H, C^7^H); 2.42 (s, 3H, CH_3_); 1.90 (s, 3H, CH_3_). ^13^C NMR (126 MHz, DMSO-*d*_6_): δ 166.70, 159.66, 158.86, 149.18, 145.49, 142.97, 139.54, 129.20, 129.10, 127.51, 127.18, 124.96, 123.85, 119.75, 118.18, 115.55, 99.53, 37.93, 37.39, 35.47, 24.54, 22.51. Anal. calcd for C_26_H_23_N_3_OS (425.16): C, 73.83; H, 5.45; N, 9.87%. Found: C, 73.68; H, 5.32; N, 9.64%.

##### 3.3.2.4 4-Cyano-3-cyanomethylsulfanyl-1,6-dimethyl-8-phenyl-7,8-dihydroisoquinoline (**5**)

**5** was synthesized by using chloroacetonitrile (**3**). m.p.: 125–127 °C. Yield: 4.6 g (85%). IR: 3054 (C–H, arom.); 2972, 2924 (C–H, aliph.); 2251 (C≡N, nonconjugated); 2212 (C≡N, conjugated). ^1^H NMR (CDCl_3_): 7.22–7.24 (m, 3H, ArH); 7.98–7.00 (d, *J* = 10, 2H, ArH); 6.61 (s, 1H, C^5^H); 4.20–4.22 (d, *J* = 10, C^8^H); 3.97–4.07 (dd, 2H, SCH_2_); 2.95–3.00 (dd, 1H, C^7^H); 2.51–2.55 (d, 1H, C^7^H), 2.36 (s, 3H, CH_3_); 1.89 (s, 3H, CH_3_). ^13^C NMR (126 MHz, DMSO-*d*_6_): δ 160.14, 155.96, 150.02, 145.89, 142.77, 129.18, 127.58, 127.29, 125.98, 118.20, 118.02, 115.11, 99.77, 37.90, 37.25, 24.64, 22.64, 15.95. Anal. calcd for C_20_H_17_N_3_S (331.11): C, 72.48; H, 5.17; N, 12.68%. Found: C, 72.67; H, 5.14; N, 12.46%.

#### 3.3.3 Cyclization of Compounds **4a**–**4c** or **5**; Synthesis of 2-Substituted-1-amino-5,8-dimethyl-6-phenyl-6,7-dihydrothieno[2,3-*c*]isoquinolines **6a**–**6c** and **7**: General Method

Compounds **4a**–**4c** or **5** (0.005 mol) were individually suspended in sodium methoxide solution (0.50 g of sodium in 30 mL methanol) and stirred at room temperature for 1 h. The yellow precipitates were individually filtered off, washed with methanol, and dried in the air to give canary crystals of compounds **6a**–**6c** and **7**.

##### 3.3.3.1 1-Amino-2-[*N*-(4-chlorophenyl)carbamoyl)-5,8-dimethyl-6-phenyl-6,7-dihydro-thieno[2,3-*c*]isoquinoline (**6a**)

**6a** was obtained by cyclization of compound **4a**. Yield: 2.2 g (95%). m.p.: 258–259 °C. IR: 3486, 3300 (NH_2_, NH); 3018 (C–H, arom.); 2920, 2866 (C–H, aliph.); 1633 (C=O, C=N); 1586 (C=C). ^1^H NMR (DMSO-*d*_6_): 9.54 (s, 1H, NH); 7.69–7.71 (d, *J* = 10, 2H, ArH); 7.33–7.35 (d, *J* = 10, 2H, ArH); 7.12–7.21 (m, 6H: C^9^H, NH_2_ and ArH); 6.68–6.70 (d, *J* = 10, 2H, ArH); 4.35–4.37 (d, *J* = 10, IH, C^6^H); 2.91–2.94 (dd, 1H, C^7^H); 2.44–2.47 (d, 1H, C^7^H); 2.28 (s, 3H, CH_3_); 1.82 (s, 3H, CH_3_). ^13^C NMR (126 MHz, DMSO-*d*_6_): δ 164.90, 158.51, 157.71, 150.22, 144.41, 142.87, 139.56, 138.49, 128.93, 128.80, 127.75, 127.67, 126.97, 125.29, 123.31, 119.27, 118.22, 98.09, 37.78, 37.04, 24.83, 22.79. Anal. calcd for C_26_H_22_ClN_3_OS (459.12): C, 67.89; H, 4.82; N, 9.13%. Found: C, 67.65; H, 4.71; N, 8.88%.

##### 3.3.3.2 1-Amino-5,8-dimethyl-6-phenyl-2-[*N*-(4-tolyl)carbamoyl)-6,7-dihydrothieno[2,3-*c*] isoquinoline (**6b**)

**6b** was obtained by cyclization of compound **4b**. Yield: 2.1 g (95%). m.p.: 245–246 °C. IR: 3449, 3304 (NH_2_, NH); 3019 (C–H, arom.); 2973, 2918 (C–H, aliph.); 1636 (C=O); 1586 (C=C). ^1^H NMR (DMSO-*d*_6_): 9.36 (s, 1H, NH); 7.52–7.54 (d, *J* = 10, 2H, ArH); 6.98–7.25 (m, 10H: C^9^H, NH_2_, and ArH); 4.35–4.37 (d, *J* = 10, IH, C^6^H); 2.91–2.94 (dd, 1H, C^7^H); 2.44–2.47 (d, 1H, C^7^H); 2.28 (s, 3H, CH_3_); 2.24 (s, 3H, CH_3_); 1.82 (s, 3H, CH_3_). ^13^C NMR (126 MHz, DMSO-*d*_6_): δ 164.77, 158.39, 157.45, 149.67, 144.21, 142.92, 139.45, 136.87, 133.03, 129.33, 128.92, 127.75, 126.96, 125.22, 122.00, 119.45, 118.27, 98.74, 37.81, 37.06, 24.83, 22.78, 21.01. Anal. calcd for C_27_H_25_N_3_OS (439.17): C, 73.77; H, 5.73; N, 9.56%. Found: C, 73.82; H, 5.69; N, 9.26%.

##### 3.3.3.3 1-Amino-5,8-dimethyl-6-phenyl-2-(*N*-phenylcarbamoyl)-6,7-dihydrothieno[2,3-*c*] isoquinoline (**6c**)

**6c** was obtained by cyclization of compound **4c**. Yield: 2.1 g (93%). m.p.: 242–243 °C. IR: 3447, 3281 (NH_2_, NH); 2960,2922 (C–H, aliph.); 1645 (C=O); 1632 (C=N). ^1^H NMR (CDCl_3_): 6.98–7.59 (m, 12H: C^9^H, NH, and ArH); 6.40 (s, 2H, NH_2_); 4.26–4.28 (d, *J* = 10, IH, C^6^H); 2.94–2.99 (dd, 1H, C^7^H); 2.49–2.53 (d, 1H, C^7^H); 2.39 (s, 3H, CH_3_); 1.87 (s, 3H, CH_3_). ^13^C NMR (126 MHz, DMSO-*d*_6_): δ 164.76, 158.37, 157.44, 149.63, 144.20, 142.93, 139.45, 136.90, 133.00, 129.33, 128.93, 127.75, 126.97, 125.22, 122.00, 119.43, 118.27, 37.78, 37.05, 24.84, 22.79, 21.01. Anal. calcd for C_26_H_23_N_3_OS (425.16): C, 73.83; H, 5.45; N, 9.87%. Found: C, 74.16; H, 5.42; N, 9.91%

##### 3.3.3.4 1-Amino-2-cyano-5,8-dimethyl-6-phenyl-6,7-dihydrothieno[2,3-*c*]isoquinoline (**7**)

**7** was obtained by cyclization of compound **5**. Yield: 1.5 g (90%). m.p.: 240–241 °C. IR: 3395, 3328, 3232 (NH_2_); 2188 (C≡N); 1640 (C=N). ^1^H NMR (DMSO-*d*_6_): 7.13–7.19 (m, 4H, C^9^H and ArH); 6.95–6.97 (t, 2H, ArH); 6.60 (s, 2H, NH_2_); 4.34–4.36 (d, *J* = 10, 1H, C^6^H); 2.87–2.92 (dd, 1H, C^7^H); 2.40–2.44 (d, 1H, C^7^H); 2.25 (s, 3H, CH_3_); 1.81 (s, 3H, CH_3_). ^13^C NMR (126 MHz, DMSO-*d*_6_): δ 159.62, 158.31, 152.62, 145.13, 142.63, 139.69, 128.94, 127.71, 127.01, 125.64, 117.95, 117.38, 116.43, 74.24, 37.65, 37.05, 24.67, 22.74. MS: *m*/*z* 331, M^+^, 15%. Anal. calcd for C_20_H_17_N_3_S (331.11): C, 72.48; H, 5.17; N, 12.68%. Found: C, 72.26; H, 5.10; N, 12.82%.

### 3.4 Preparing Studied Surface and Tested Media

The mild steel (MS) specimen composition is (wt %) Fe 98%. For the electrochemical studies, the MS specimens were divided into 1 × 1 × 1 cm^3^. The surfaces of all tested specimens were polished with different grades of emery polishing papers such as 1200 and 1400, then degreased with acetone and finally dried. The corrosive solutions were prepared by analytical grade 97% H_2_SO_4_ (Sigma-Aldrich Laborchemikalien, German) with dilution by bidistilled water.

### 3.5 Preparation of Corrosive and Inhibitor Solutions

The inhibitors (He1-Ph-Cl, He2-Ph-CH_3_, He3-Ph, and He4-CN) were prepared by weighing 0.05 g of the tested inhibitors and dissolving it into 100 cm^3^ of 1.0 M H_2_SO_4_ to obtain 500 ppm of each inhibitor that was diluted to 100 and 200 ppm to execute the experimental task.

### 3.6 Electrochemical Techniques

The potentiodynamic method used in this study includes open circuit potential (OCP) for immersion MS electrode potential (*E*_im_) in the blank solution without and with inhibitor concentrations to obtain a steady-state potential (*E*_ss_), which is near the corrosion potential (*E*_corr_ ≈ *E*_ocp_). Potentiodynamic polarization (PP) records parameters such as corrosion potential (*E*_corr_, mV), corrosion current density (*I*_corr_, μA/cm^2^), corrosion rate (CR, millimeter per year, mpy), inhibition efficiency percentage (IE%), and surface coverage (θ = IE%/100). OCP is performed using a reference electrode as SEC and working MS, but in PP experiments, a counter electrode (Pt wire) is added with the obvious OCP electrodes. OCP and PP curves were performed with an EG&G potentiostat/galvanostat instrument, model 273A. TF was scanned at ±250 mV vs *E*_corr≈ocp_ with a rate of scan of 0.3 mV/s. CR and IE% for the tested inhibitors were calculated from *I*_corr_ mathematically according to eqs 3 and 4, respectively.^37^
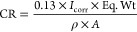
3where CR is the corrosion rate (mpy); *I*_corr_ is the corrosion current density (μA/cm^2^), which records the current value at which the corrosion process takes place; Eq. Wt. is the equivalent weight of the metal (gm/eq) equal to 55.8 atomic mass; *A* is the area (cm^2^) immersed in tested solutions; ρ is the density (gm/cm^3^) equal to 7.874 g/cm^3^; and 0.13 is the metric and time conversion factor.
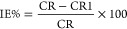
4CR and CR1 are the corrosion rates without and with inhibitors, respectively.

### 3.7 Quantum Calculations

Quantum calculations introduce some parameters related to the reactivity and stability of tested inhibitors, such as the energy gap (EG), ionization potential (*I*), chemical potential (μ), global hardness (η), global softness (σ), softness (*S*), and the fraction of electrons transferred (Δ*N*). eqs 5–11) summarize the studied quantum chemical descriptors shown as follows:^38,39^
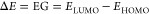
5
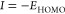
6
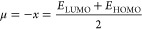
7
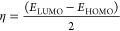
8
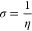
9
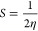
10
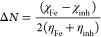
11

## 4 Conclusions

Four dihydrothienoisoquinoline (DHTIIQ) derivatives were successfully synthesized in this article, and their structures were studied using elemental and spectral studies. The photophysical properties of the synthesized DHTIIQs were investigated, and they have good luminescence properties. In a 1.0 M sulfuric acid solution, DHTIIQ derivatives were tested as anticorrosion agents for mild steel. The findings demonstrate that all examined compounds have a high inhibition ratio, reaching 95% for compound He1-Ph-Cl, following the pattern He1-Ph-Cl > He2-Ph-CH3 > He3-CN > He4-Ph as concentration increases. The Tafel polarization plots show that these compounds are mixed-type inhibitors, and the adsorption isotherm follows the Langmuir isotherm. The free energy recorded between −20.6 and −19.6 kJ/mol indicates the presence of a physisorption isotherm, and quantum calculation using the Gaussian 09 program yielded results that were consistent with the experimental results. In addition, SEM images of mild steel with and without inhibitors were studied. Tissue engineering, bioimaging, sensors/biosensors, smart labeling, and anticounterfeiting are ideal options for the synthesized DHTIIQs, promising for making corrosion inhibitors.
