# Supplementary material for: Exploration of Dihydrothieno[2,3-c] Isoquinolines As Luminescent Materials and Corrosion Inhibitors
Source: ACS Omega. 2022 Oct 19;7(43):38389–99. doi: 10.1021/acsomega.2c03361 (PMC9631879; doi:10.1021/acsomega.2c03361)
Supplement: Supplementary file 1 — ao2c03361_si_001.pdf [file ao2c03361_si_001.pdf]

Supporting Information

Exploration of Dihydrothieno[2,3-*c*] isoquinolines as Luminescent Materials and Corrosion Inhibitors

Islam S. Marae,[a] Mahmoud H. Mahross,[b] Badriah S. Al-Farhan,[c] Mohamed Abdel-Hakim,[b*] Etify A. Bakhite,[a*] Marwa M. Sayed[d]

Figure S1: [a] FT-IR and **[b]** ^{1}H NMR of the prepared compound **1:**

[a]

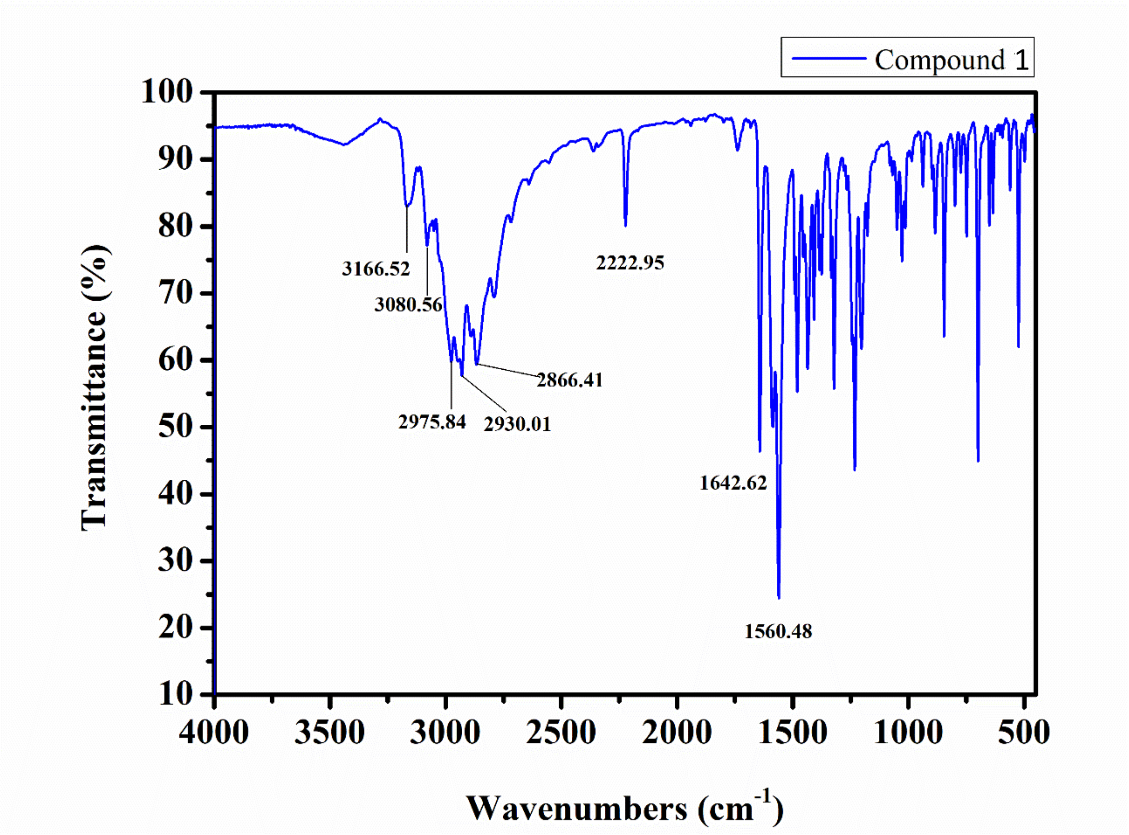

[b]

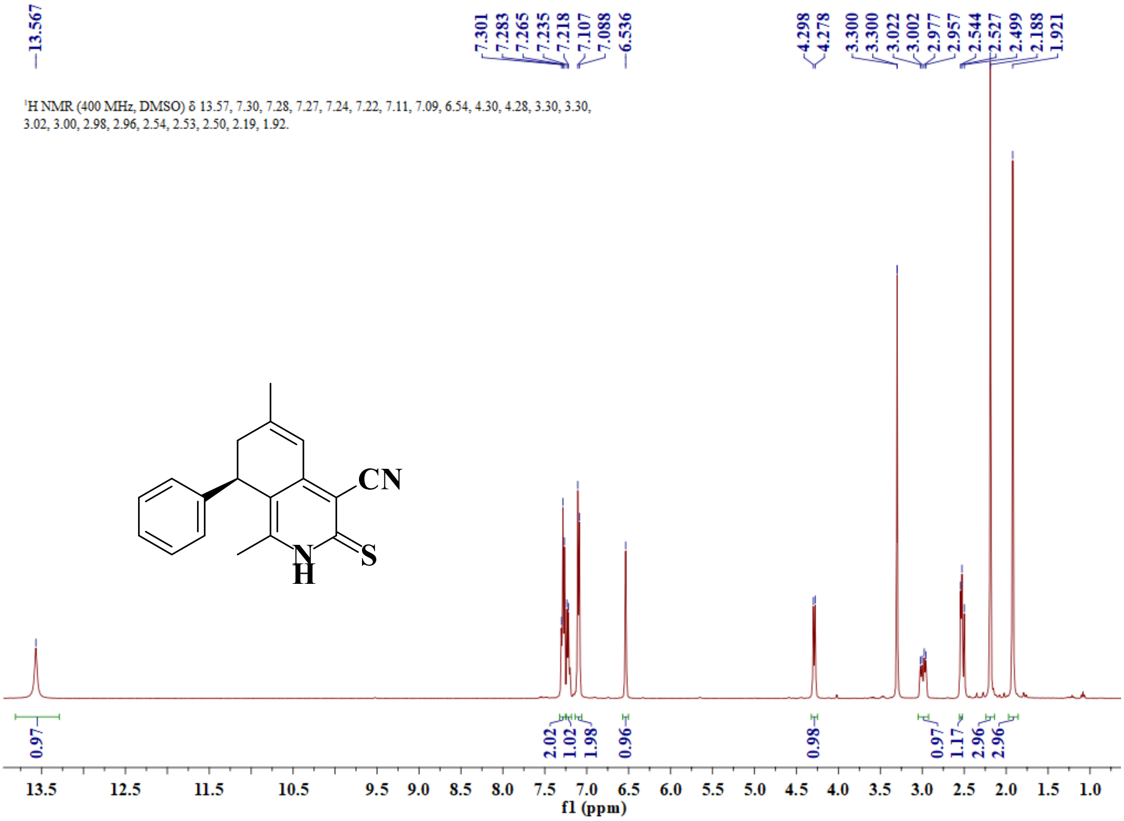

Figure S2: [a] FT-IR and [b] ^{1}H NMR [c] ^{13}C NMR of the prepared compound **4a**

[a]

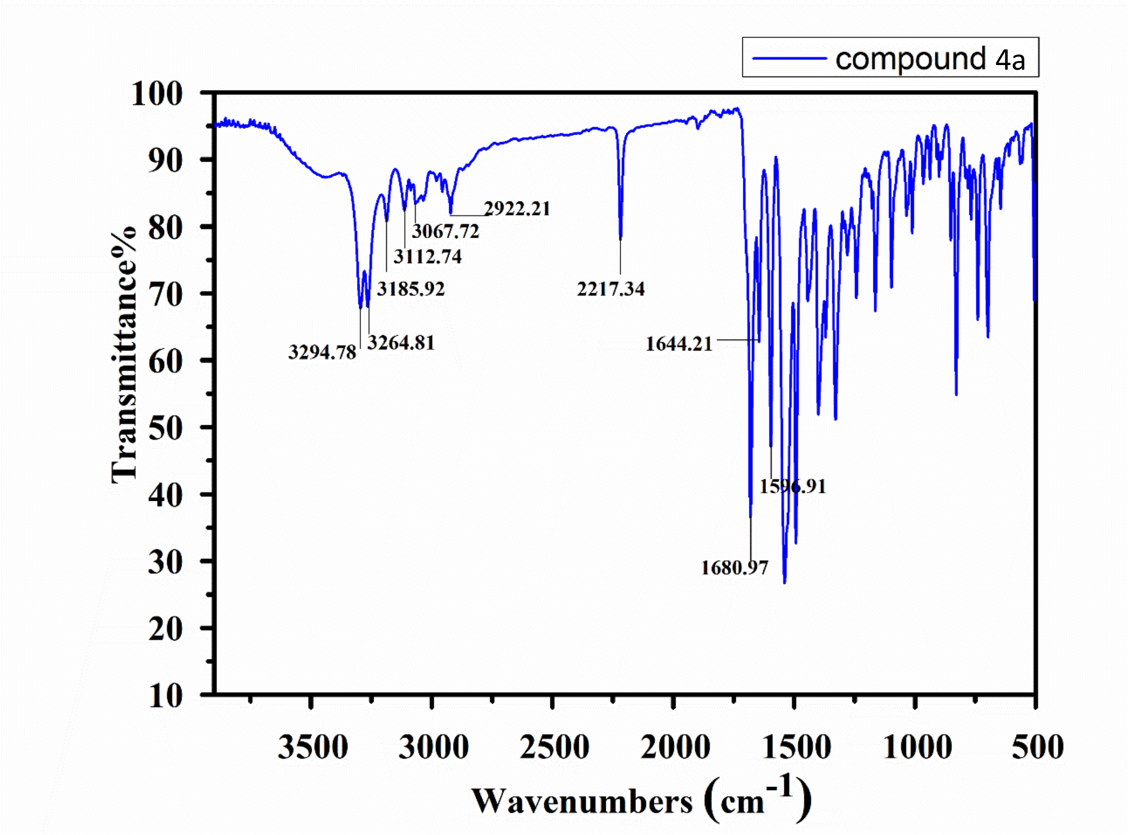

[b]

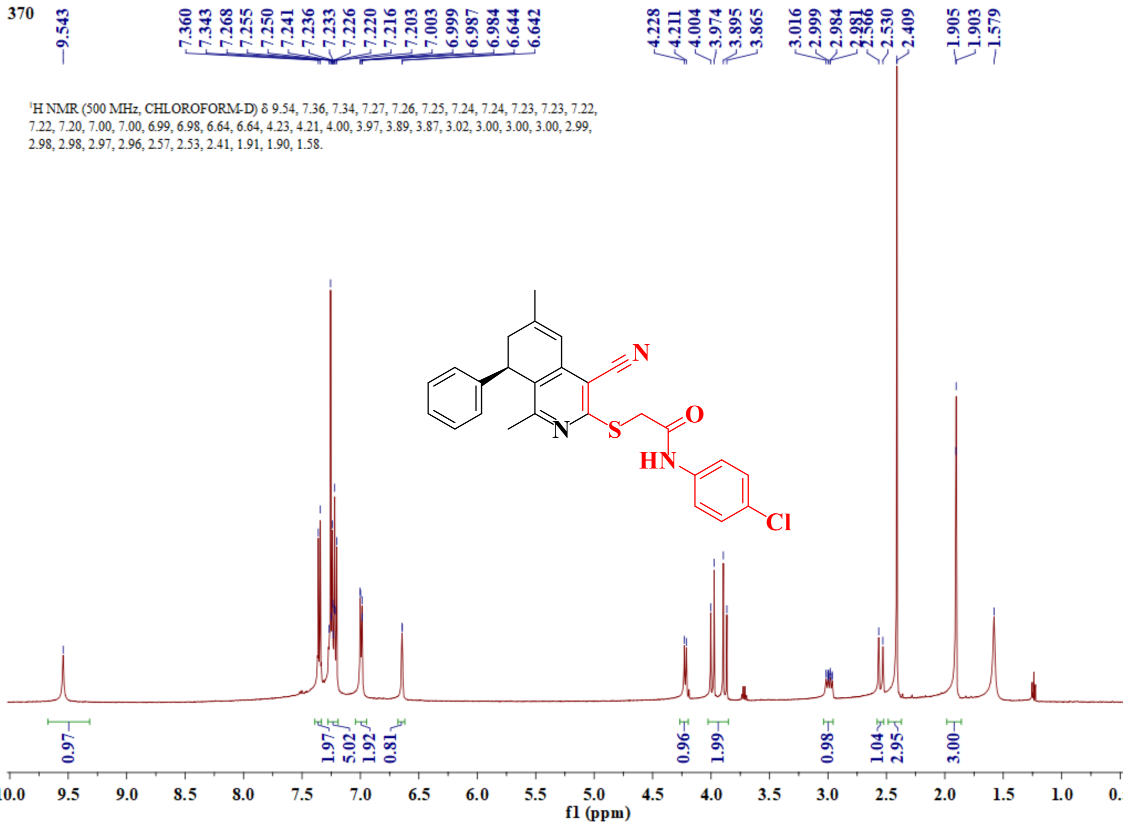

[c]

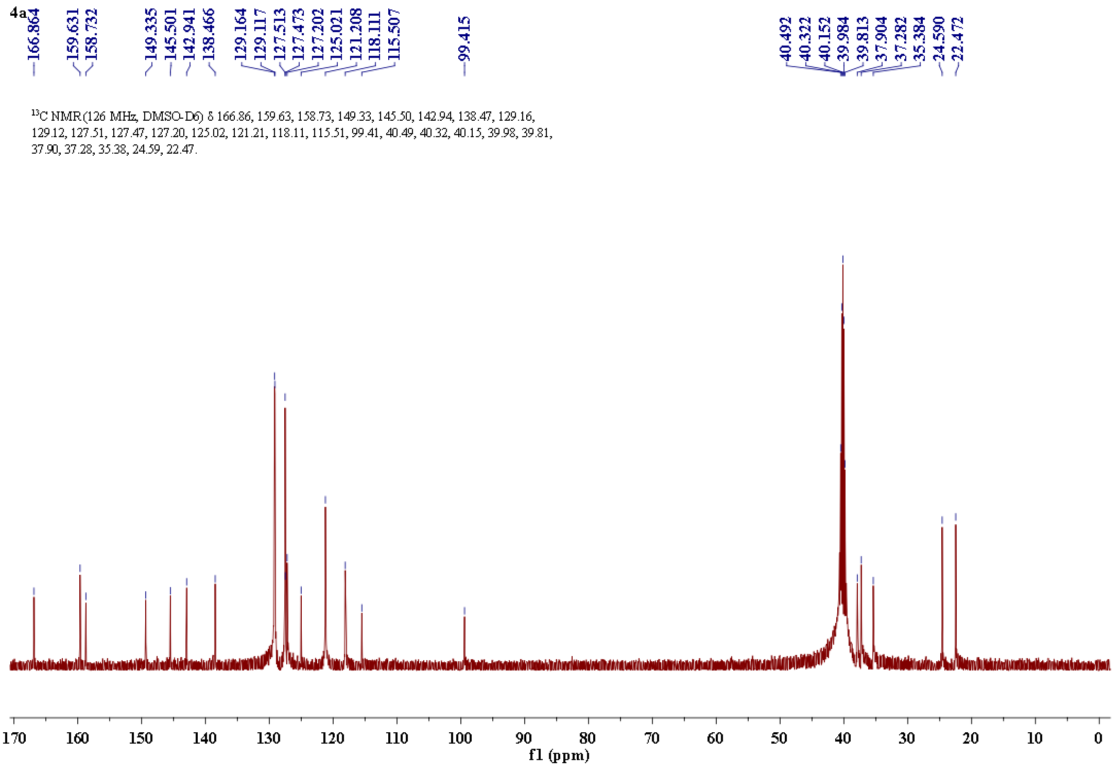

^{13}C NMR (126 MHz, DMSO-D6) δ 166.86, 159.63, 158.73, 149.33, 145.50, 142.94, 138.47, 129.16, 129.12, 127.51, 127.47, 127.20, 125.02, 121.21, 118.11, 115.51, 99.41, 40.49, 40.32, 40.15, 39.98, 39.81, 37.90, 37.28, 35.38, 24.59, 22.47.

Figure S3: [a] FT-IR and **[b]** ^{1}H NMR [c] ^{13}C NMR of the prepared compound **4b**

[a]

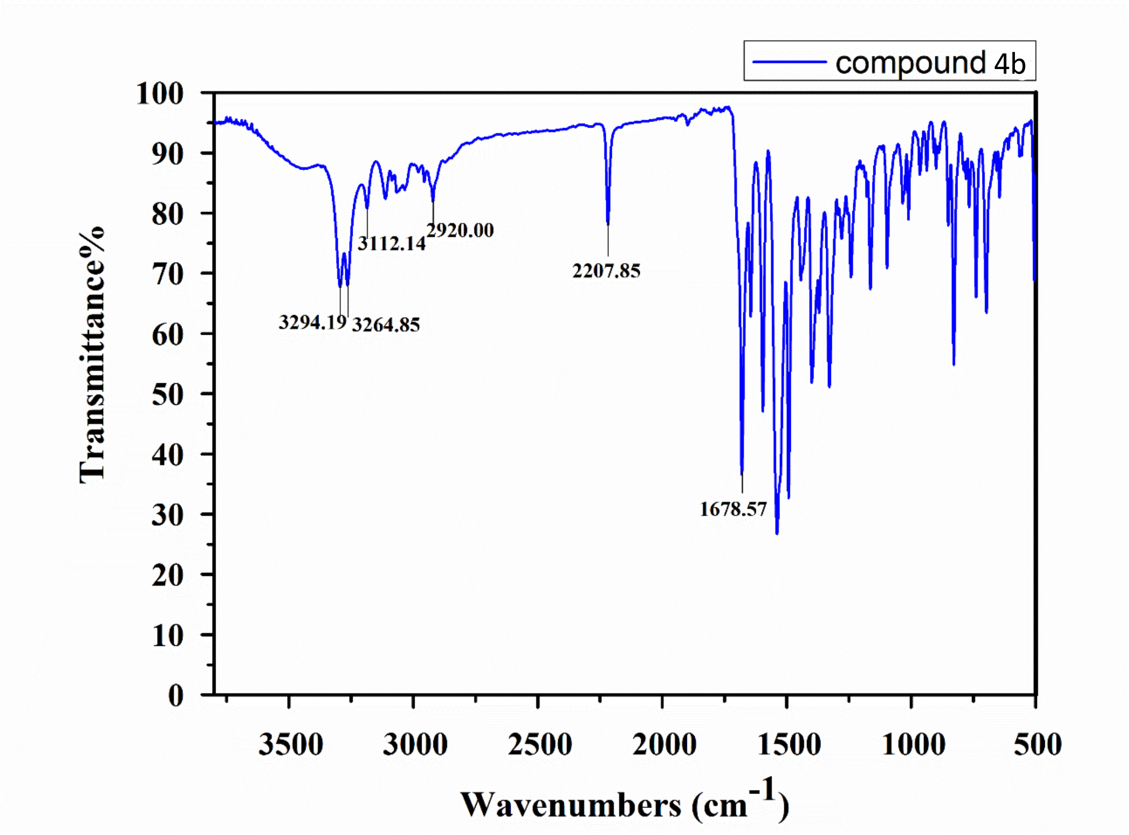

[b]

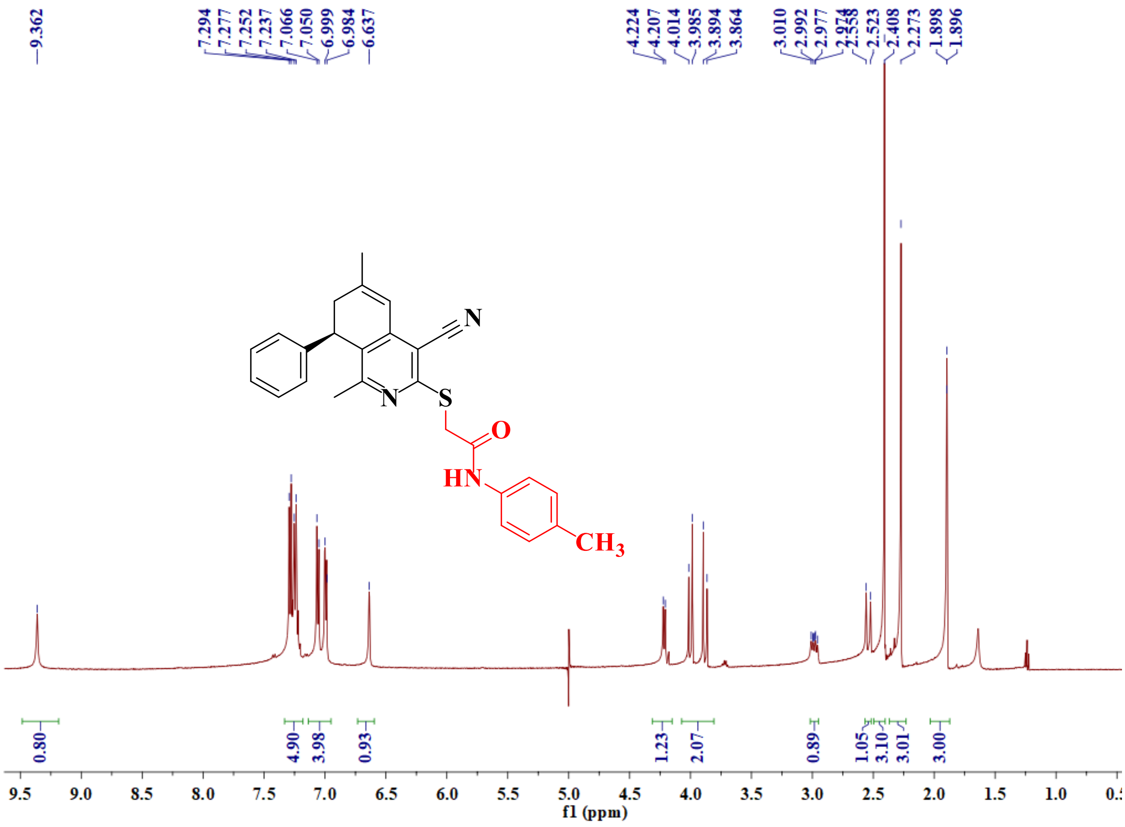

[c]

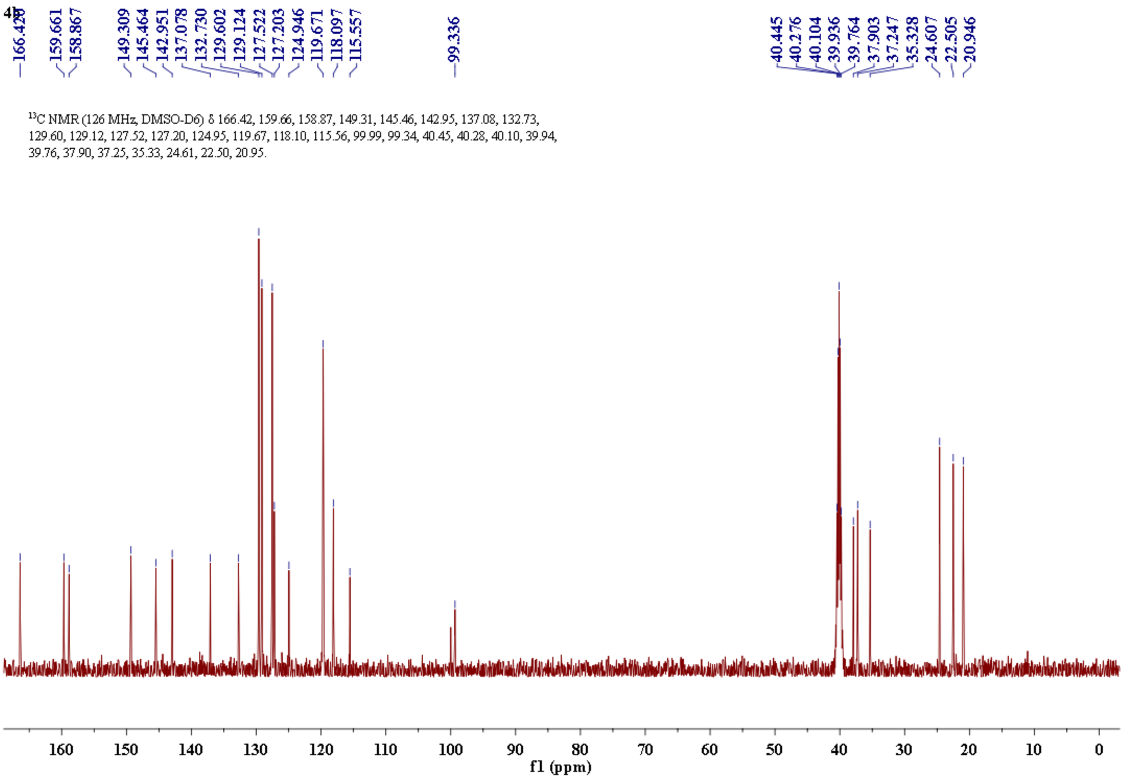
4b
166.420
159.661
158.867
149.309
145.464
142.951
137.078
132.730
129.602
129.124
127.522
127.203
124.946
119.671
118.097
115.557
99.336
40.445
40.276
40.104
39.936
39.764
37.903
37.247
35.328
24.607
22.505
20.946
^{13}C NMR (126 MHz, DMSO-D6) δ 166.42, 159.66, 158.87, 149.31, 145.46, 142.95, 137.08, 132.73, 129.60, 129.12, 127.52, 127.20, 124.95, 119.67, 118.10, 115.56, 99.99, 99.34, 40.45, 40.28, 40.10, 39.94, 39.76, 37.90, 37.25, 35.33, 24.61, 22.50, 20.95.
160
150
140
130
120
110
100
90
80
70
60
50
40
30
20
10
0
f1 (ppm)

Figure S4: [a] FT IR and **[b]** ^{1}H NMR [c] ^{13}C NMR of the prepared compound **4c**

[a]

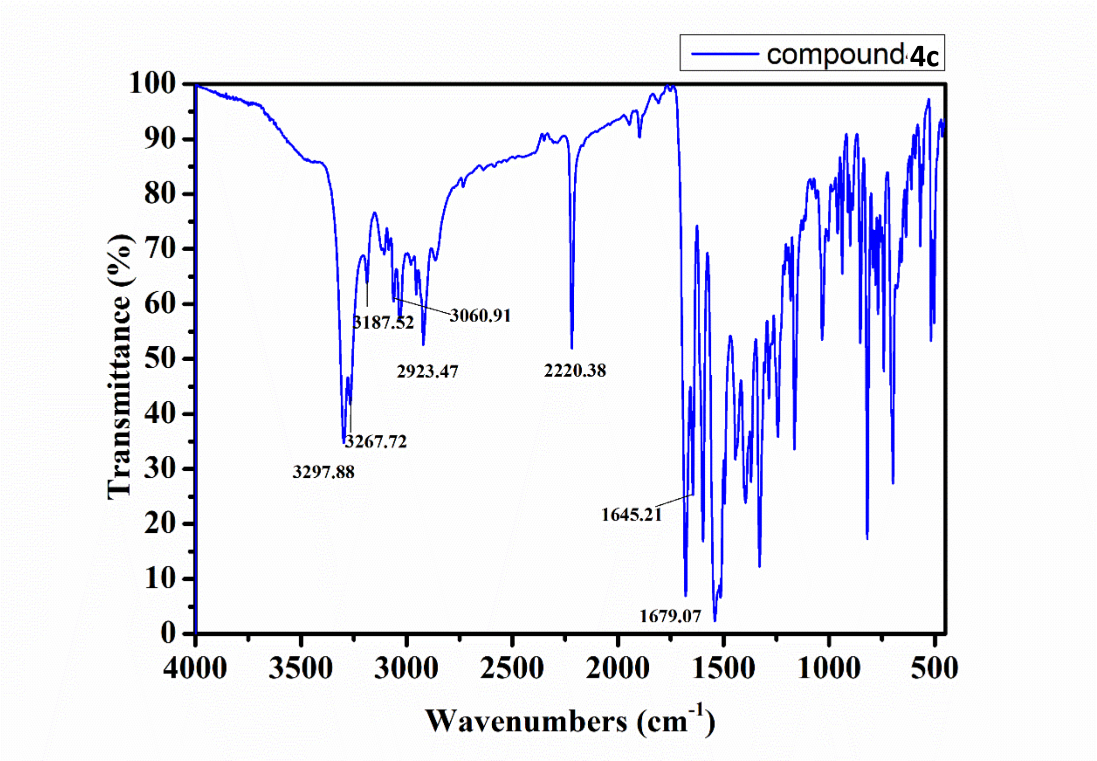

[b]

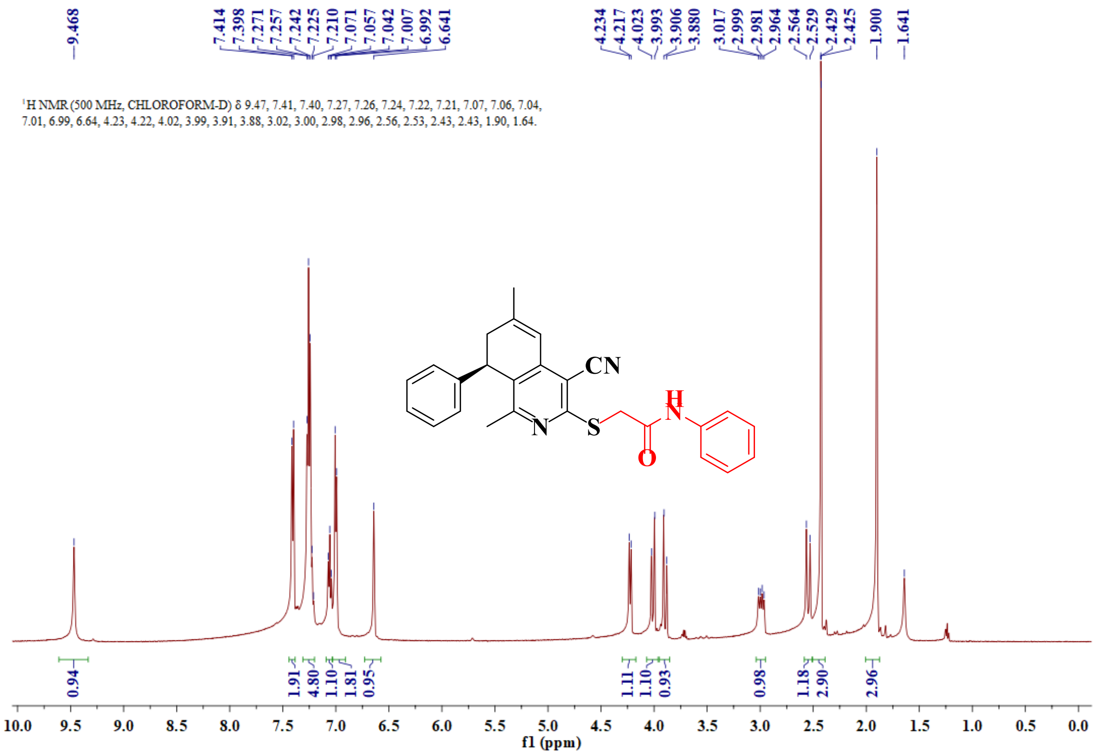

[c]

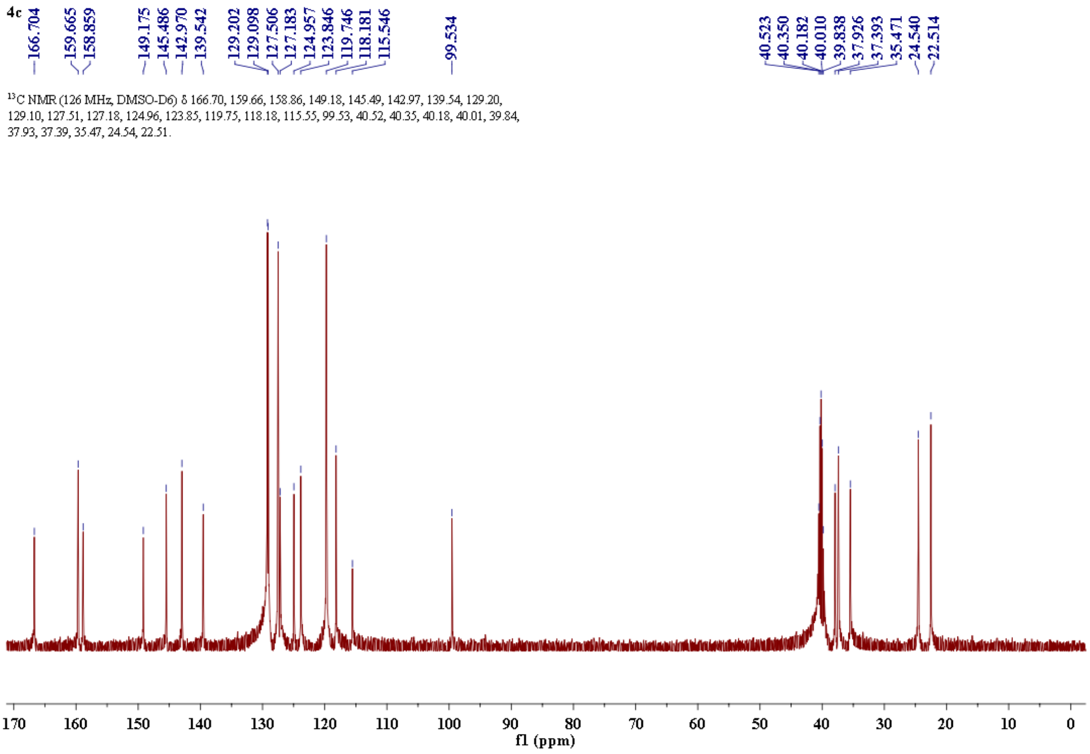

^{13}C NMR (126 MHz, DMSO-D6) δ 166.70, 159.66, 158.86, 149.18, 145.49, 142.97, 139.54, 129.20, 129.10, 127.51, 127.18, 124.96, 123.85, 119.75, 118.18, 115.55, 99.53, 40.52, 40.35, 40.18, 40.01, 39.84, 37.93, 37.39, 35.47, 24.54, 22.51.

Figure S5: [a] FT-IR and **[b]** ^{1}H NMR [c] ^{13}C NMR of the prepared compound **5**

[a]

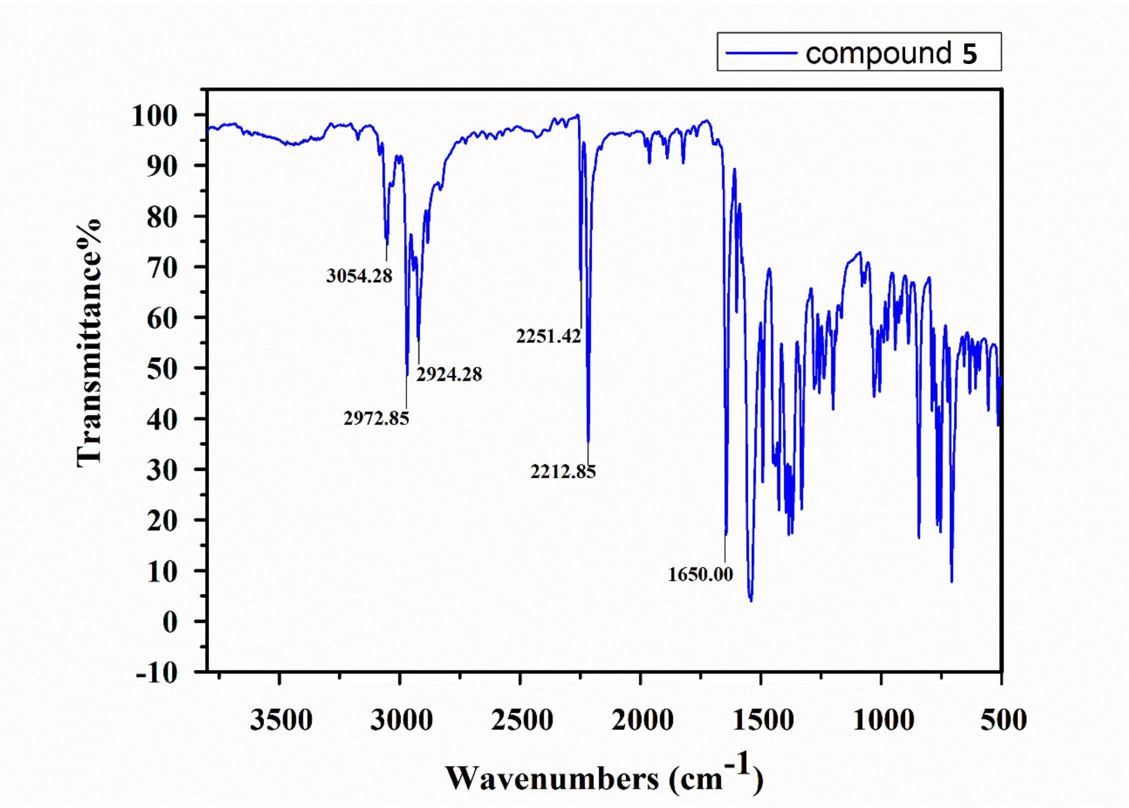

[b]

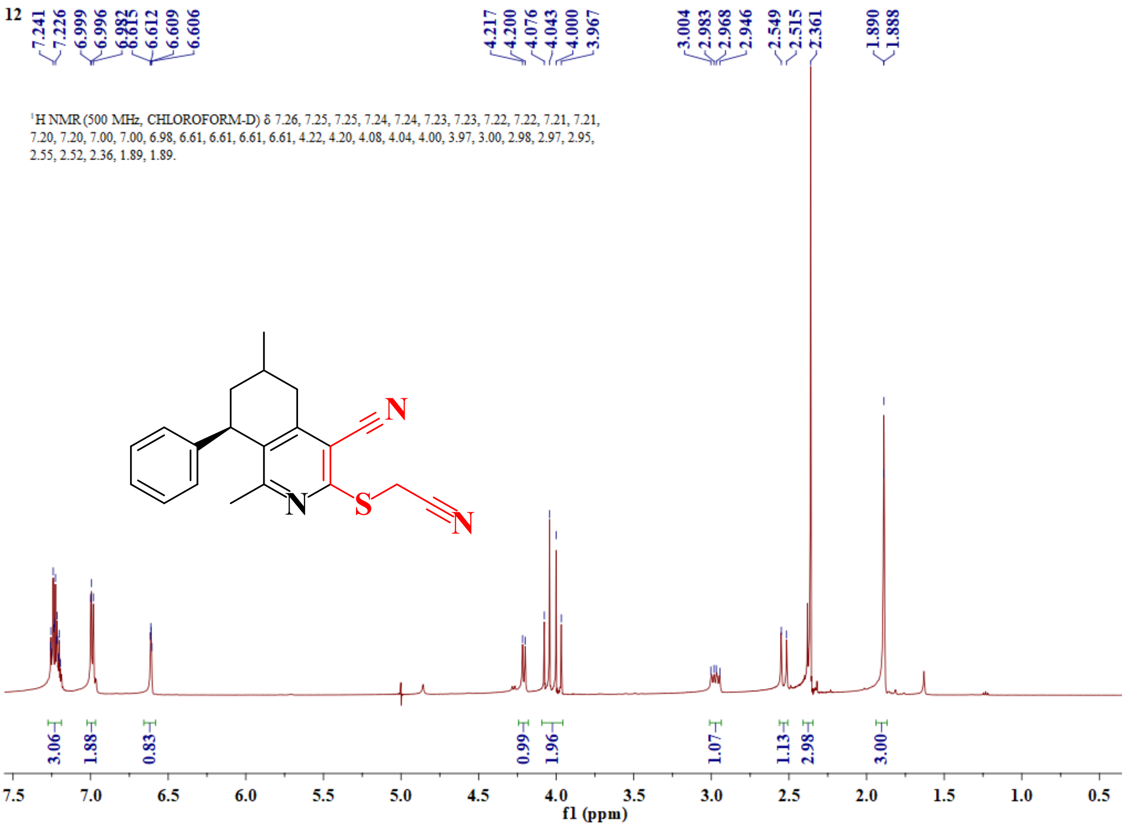

[c]

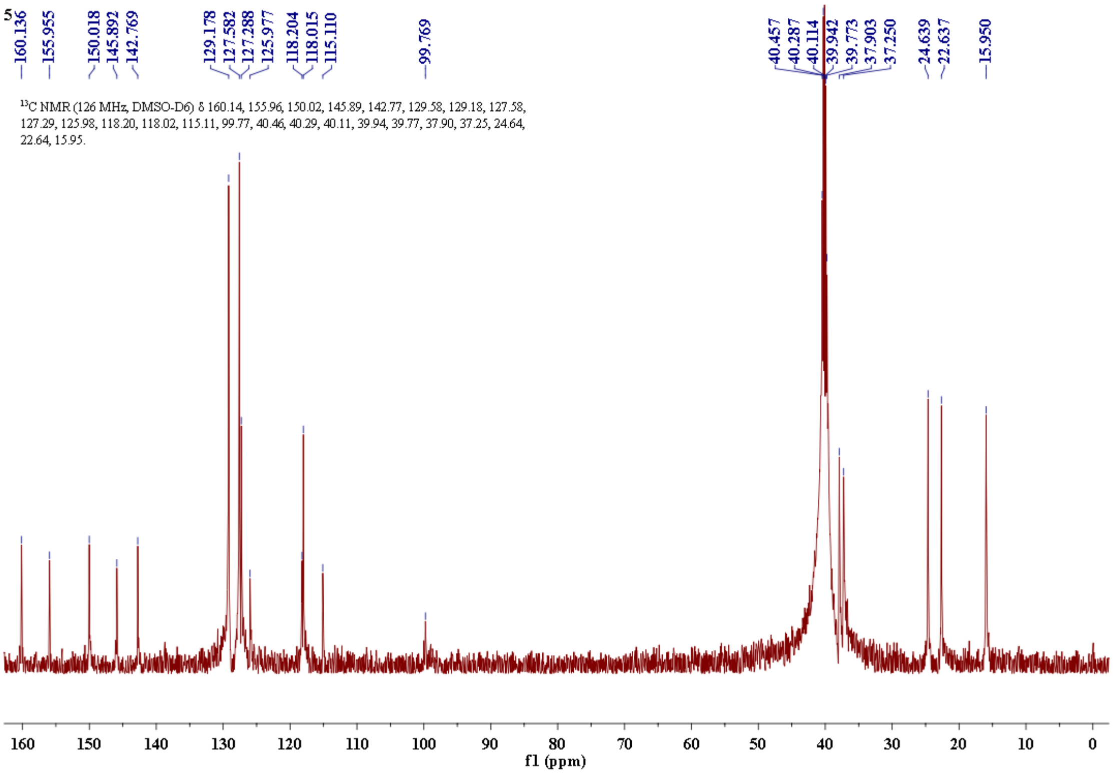
5
160.136
155.955
150.018
145.892
142.769
129.178
127.582
127.288
125.977
118.204
118.015
115.110
99.769
40.457
40.287
40.114
39.942
39.773
37.903
37.250
24.639
22.637
15.950
^{13}C NMR (126 MHz, DMSO-D6) δ 160.14, 155.96, 150.02, 145.89, 142.77, 129.58, 129.18, 127.58, 127.29, 125.98, 118.20, 118.02, 115.11, 99.77, 40.46, 40.29, 40.11, 39.94, 39.77, 37.90, 37.25, 24.64, 22.64, 15.95.
160
150
140
130
120
110
100
90
80
70
60
50
40
30
20
10
0
f1 (ppm)

Figure S6: [a] FT-IR and **[b]** ^{1}H NMR [c] ^{13}C NMR of the prepared compound **6a:**

[a]

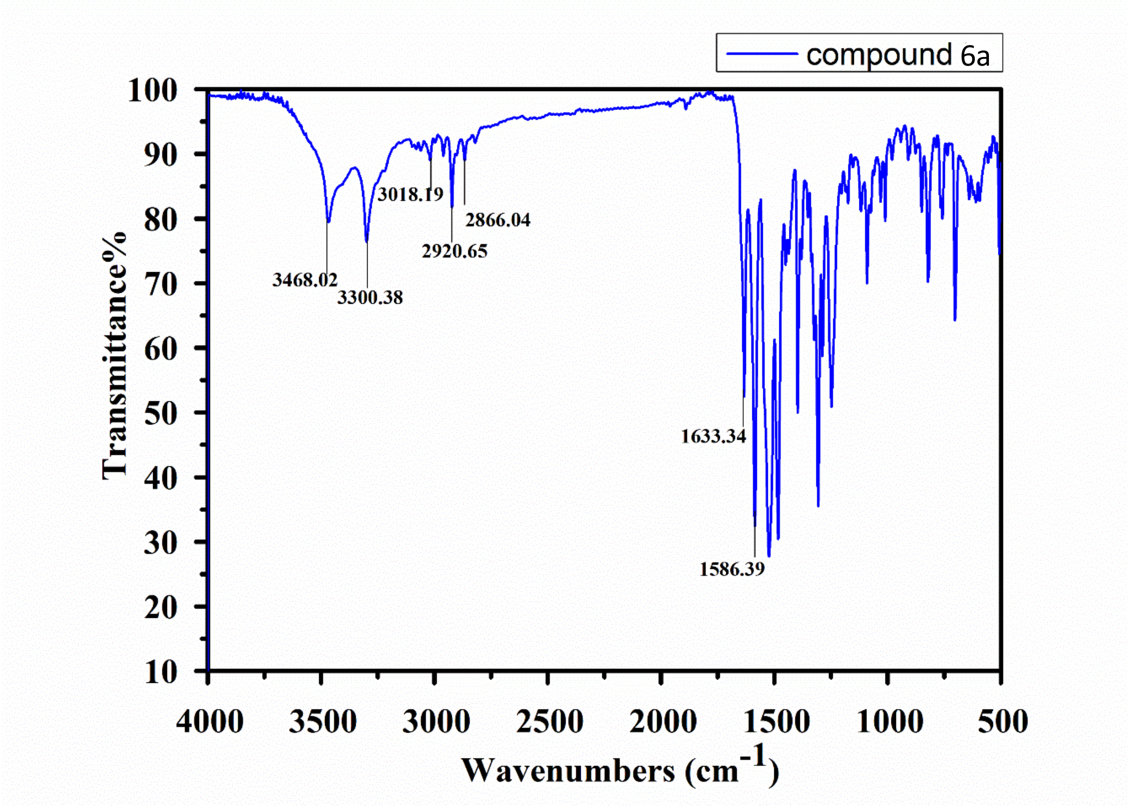

[b]

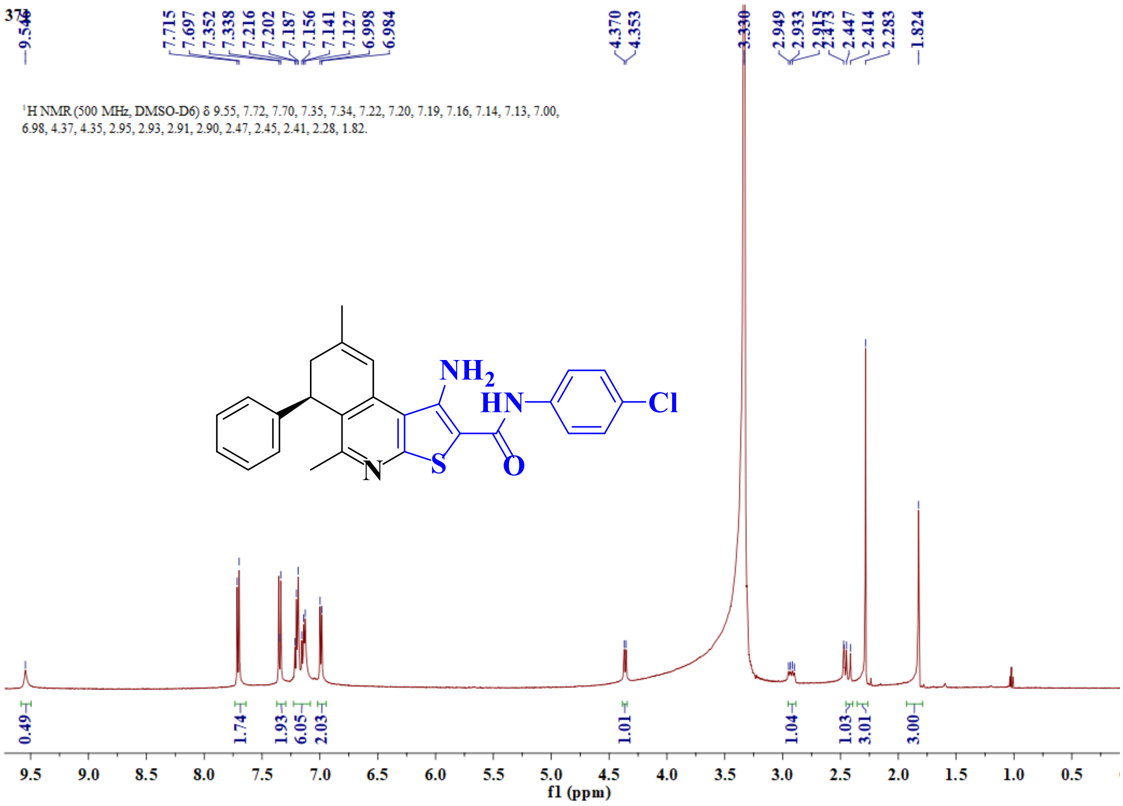

[c]

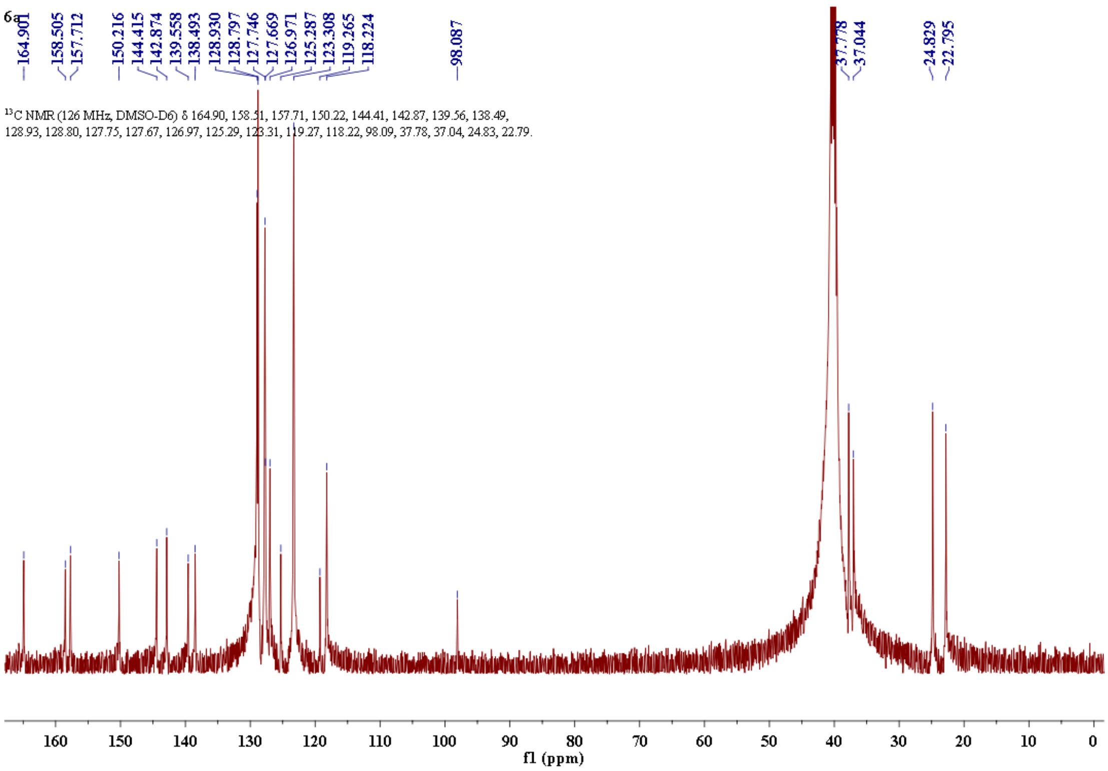
6a
164.901
158.505
157.712
150.216
144.415
142.874
139.558
138.493
128.930
128.797
127.746
127.669
126.971
125.287
123.308
119.265
118.224
98.087
37.778
37.044
24.829
22.795
^{13}C NMR (126 MHz, DMSO-D6) δ 164.90, 158.51, 157.71, 150.22, 144.41, 142.87, 139.56, 138.49, 128.93, 128.80, 127.75, 127.67, 126.97, 125.29, 123.31, 119.27, 118.22, 98.09, 37.78, 37.04, 24.83, 22.79.
160
150
140
130
120
110
100
90
80
70
60
50
40
30
20
10
0
f1 (ppm)

Figure S7: [a] FT-IR **[b]** ^{1}H NMR and [c] ^{13}C NMR of the prepared compound **6b**

[a]

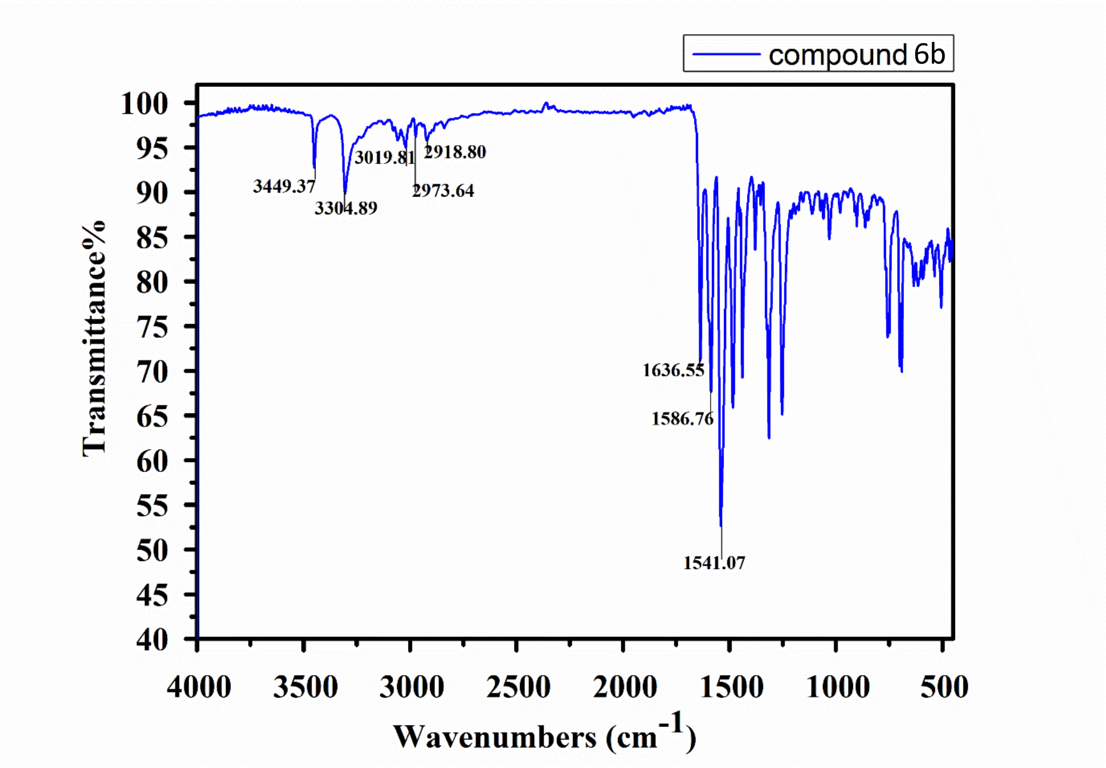

[b]

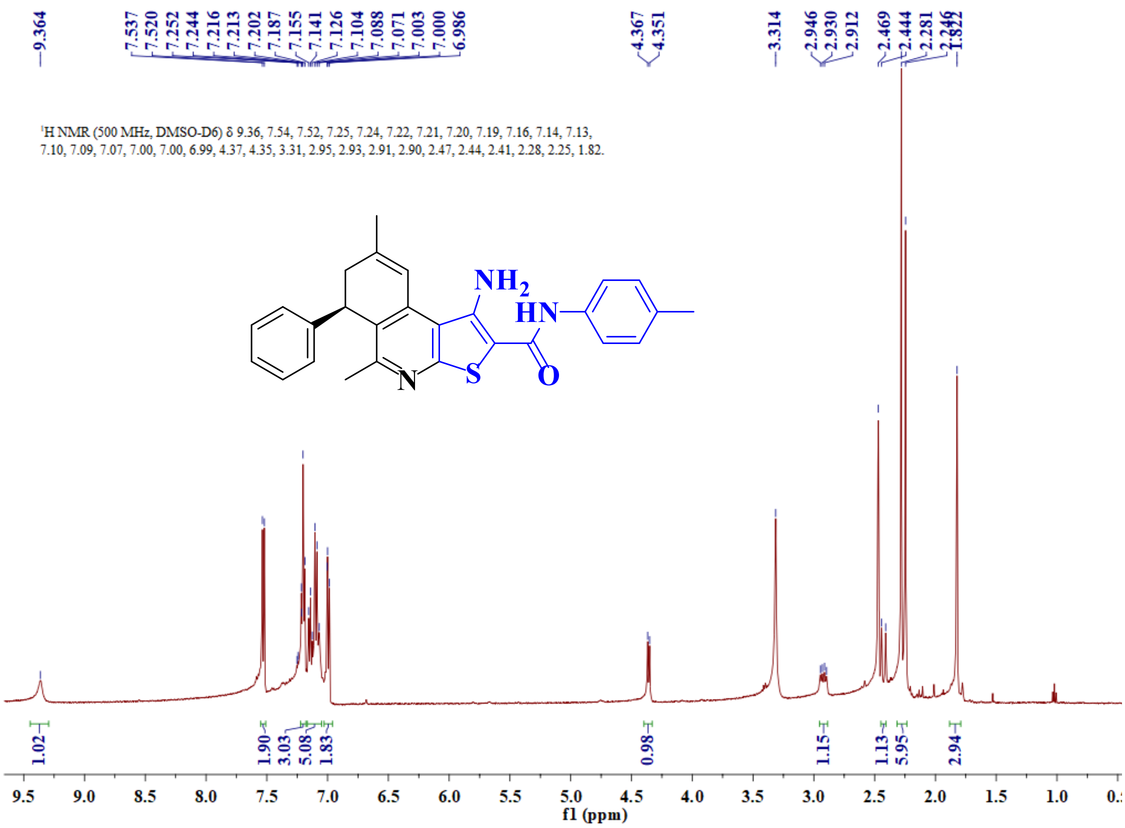

[c]

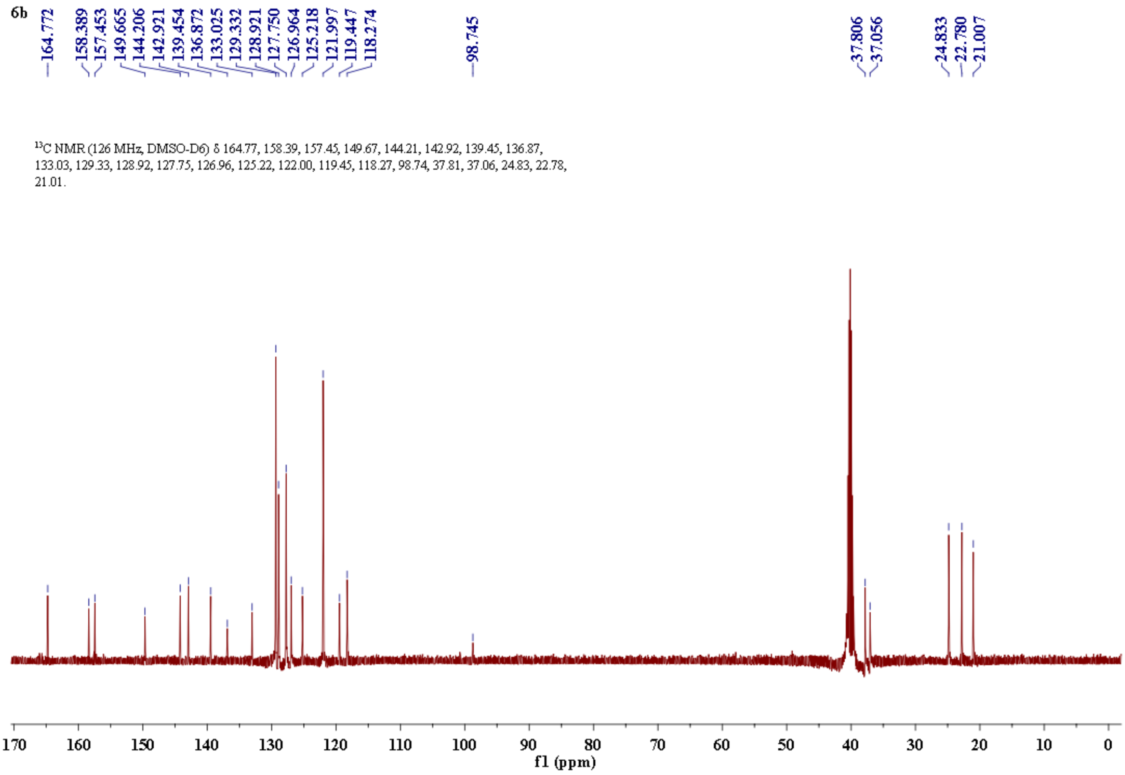
6b
164.772
158.389
157.453
149.665
144.206
142.921
139.454
136.872
133.025
129.332
128.921
127.750
126.964
125.218
121.997
119.447
118.274
98.745
37.806
37.056
24.833
22.780
21.007
^{13}C NMR (126 MHz, DMSO-D6) δ 164.77, 158.39, 157.45, 149.67, 144.21, 142.92, 139.45, 136.87, 133.03, 129.33, 128.92, 127.75, 126.96, 125.22, 122.00, 119.45, 118.27, 98.74, 37.81, 37.06, 24.83, 22.78, 21.01.
170
160
150
140
130
120
110
100
90
80
70
60
50
40
30
20
10
0
f1 (ppm)

Figure S8: [a] FT-IR **[b]** ^{1}HNMR [c] and ^{13}C NMR of the prepared compound **6c**

[a]

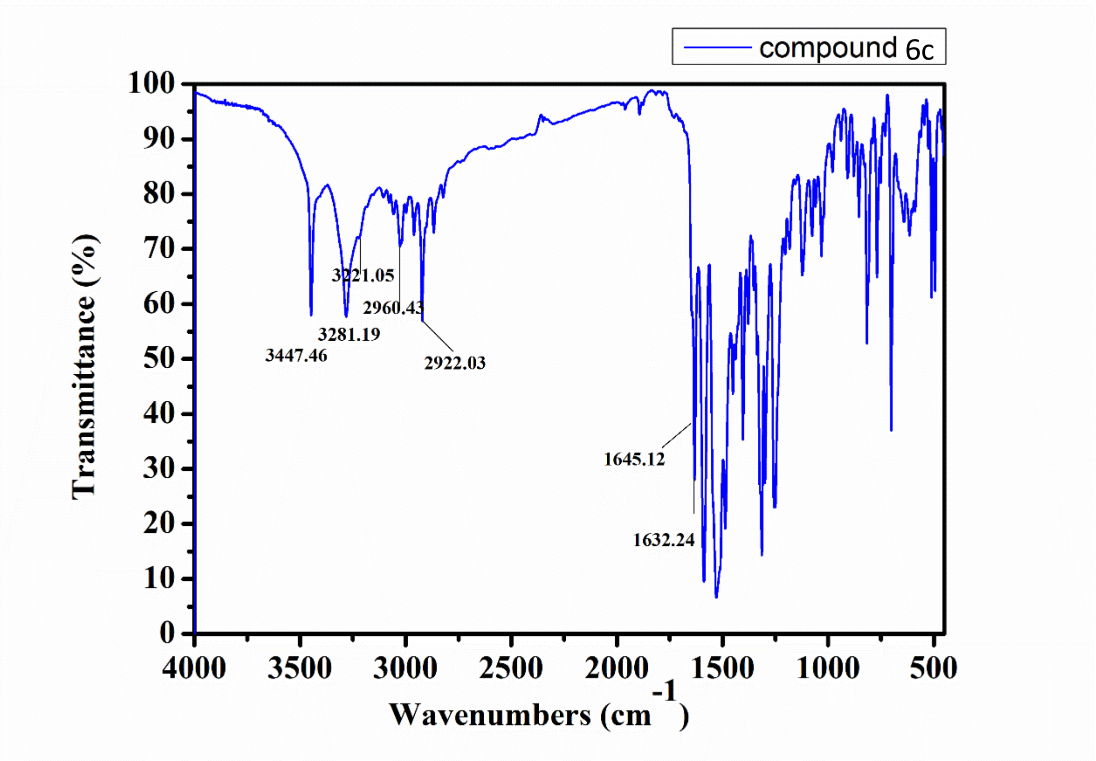

[b]

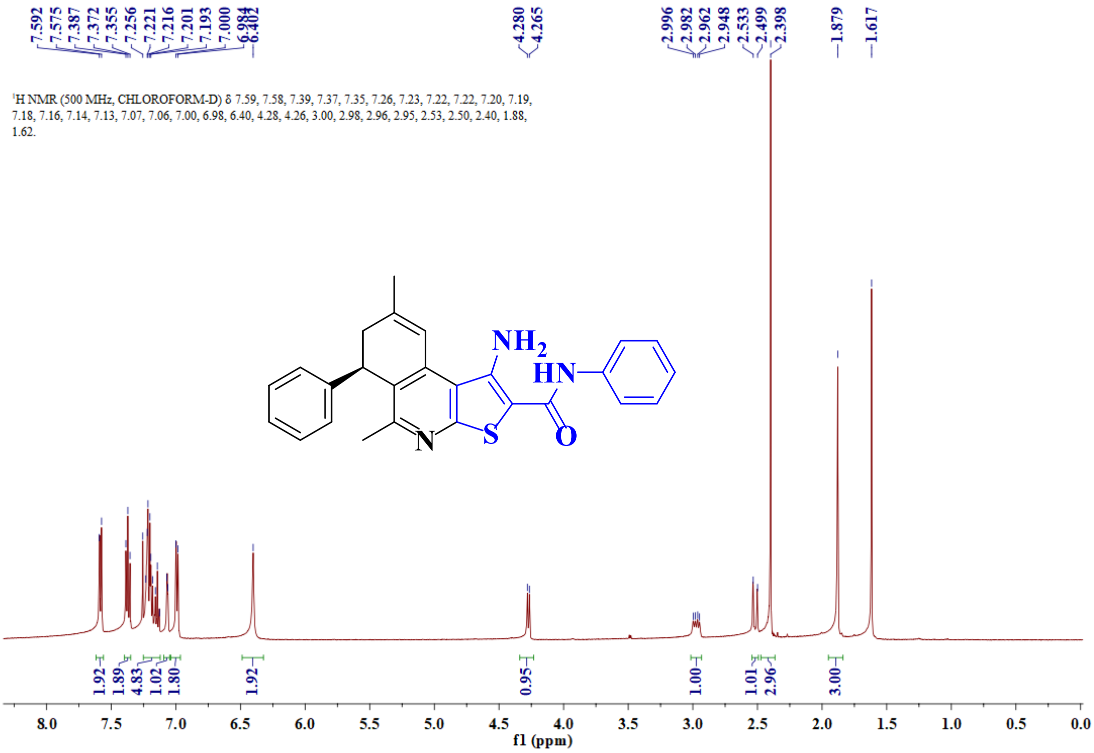

[c]

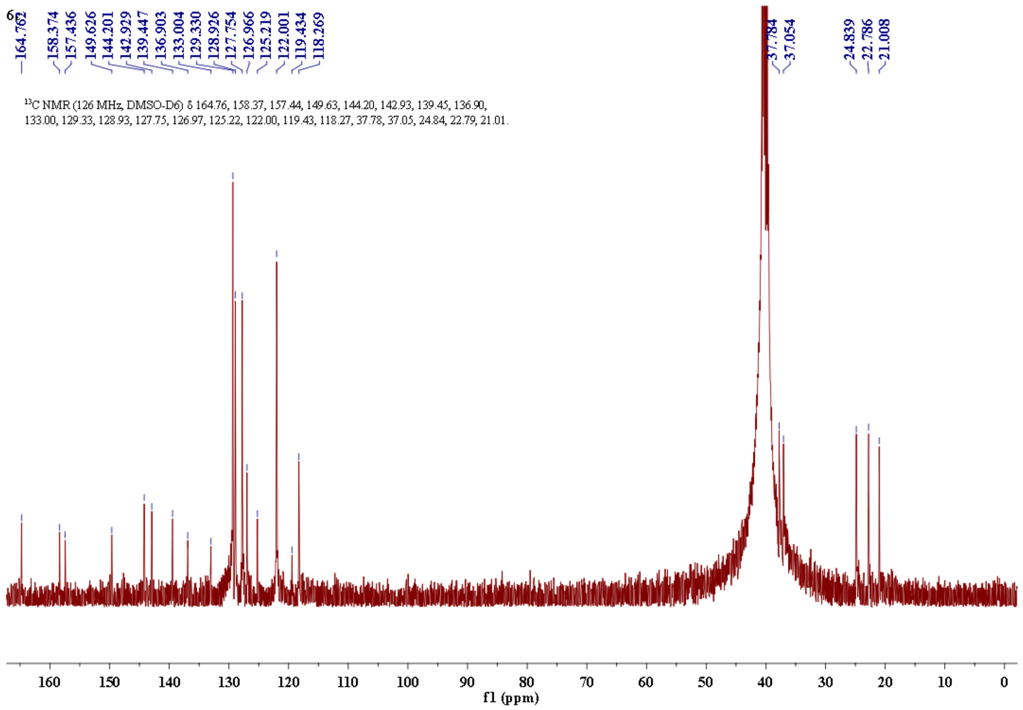
6c
164.762
158.374
157.436
149.626
144.201
142.929
139.447
136.903
133.004
129.330
128.926
127.754
126.966
125.219
122.001
119.434
118.269
37.784
37.054
24.839
22.786
21.008
13C NMR (126 MHz, DMSO-D6) δ 164.76, 158.37, 157.44, 149.63, 144.20, 142.93, 139.45, 136.90, 133.00, 129.33, 128.93, 127.75, 126.97, 125.22, 122.00, 119.43, 118.27, 37.78, 37.05, 24.84, 22.79, 21.01.
160
150
140
130
120
110
100
90
80
70
60
50
40
30
20
10
0
f1 (ppm)

Figure S9: [a] FT-IR **[b]** ^{1}HNMR [c] and ^{13}C NMR of the prepared compound **7:**

[a]

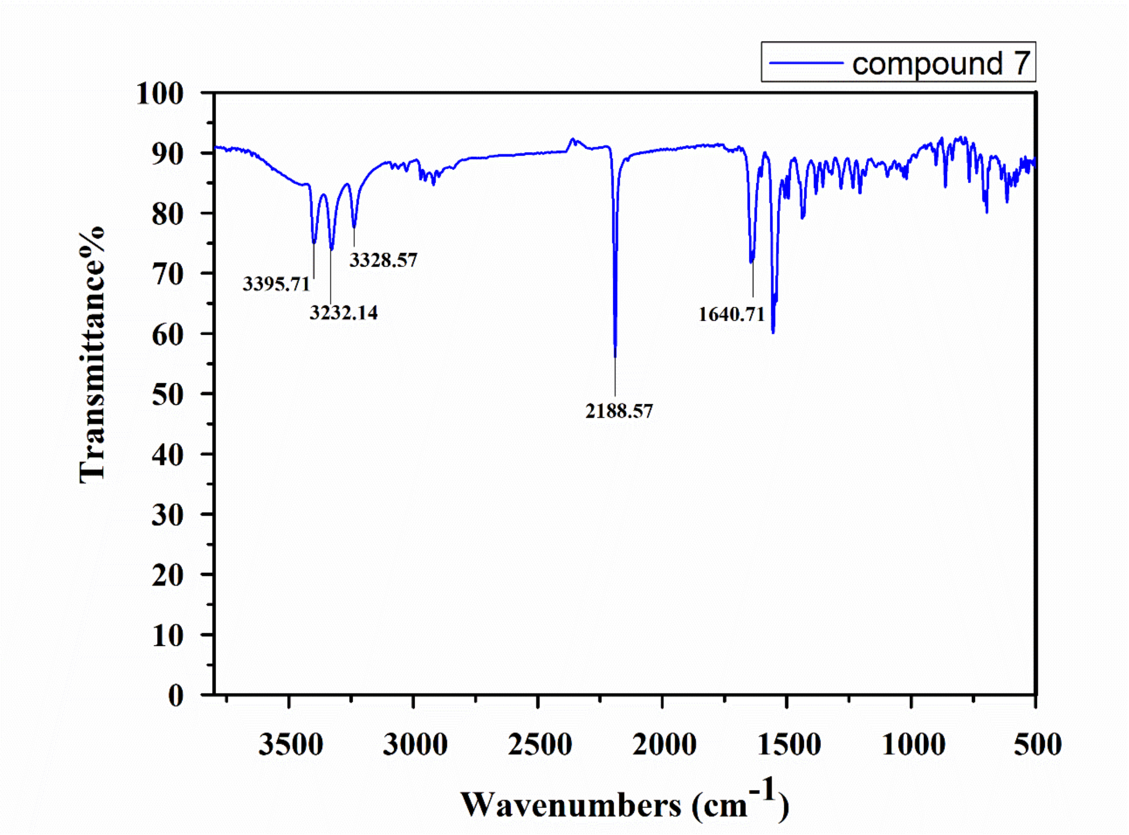

[b]

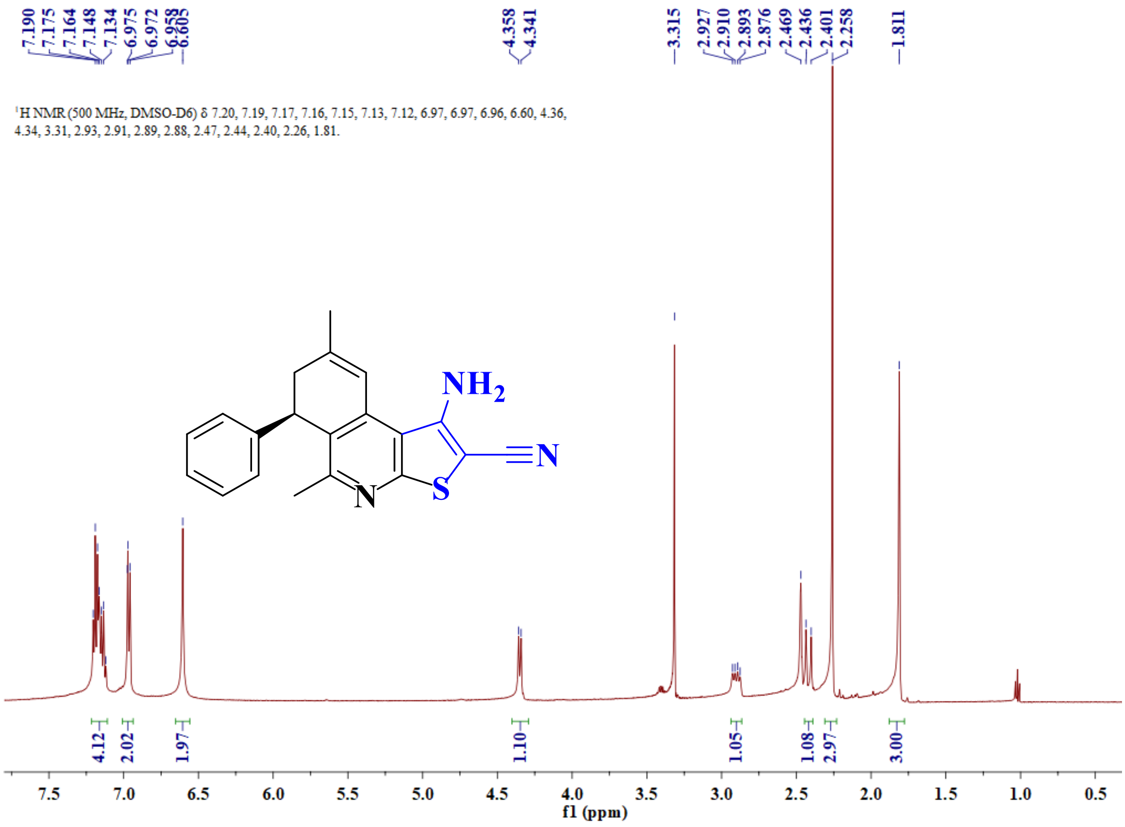

[c]

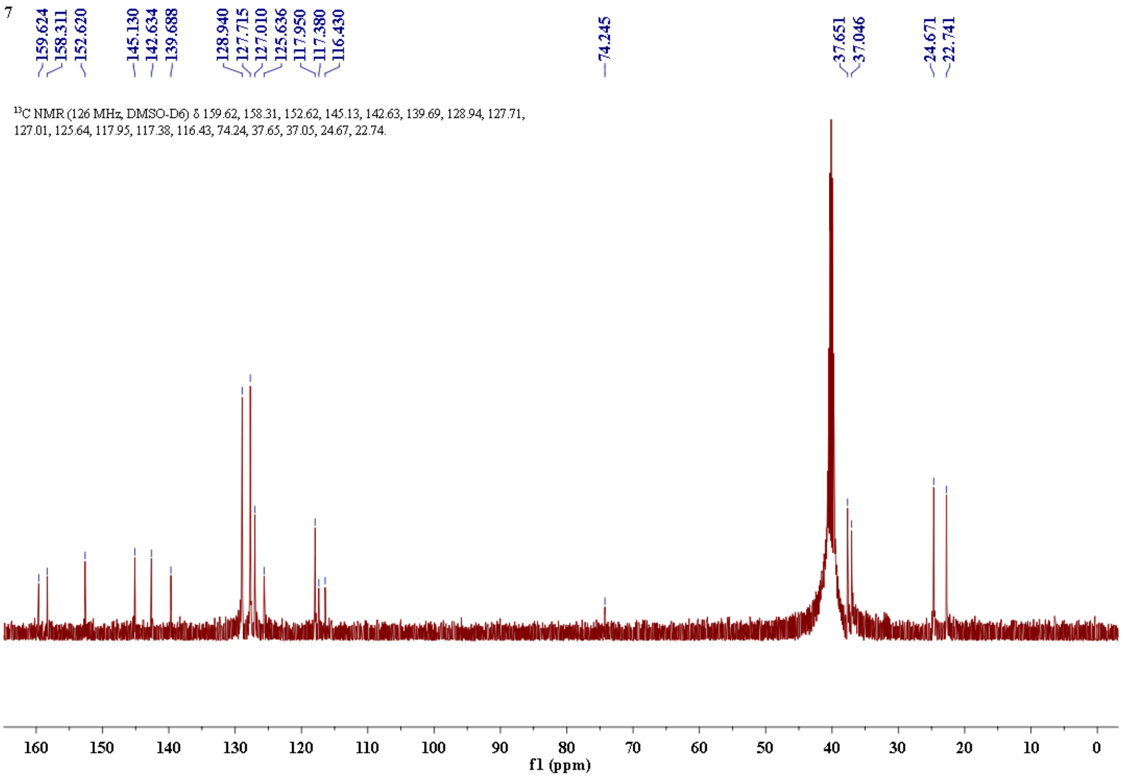
7
159.624
158.311
152.620
145.130
142.634
139.688
128.940
127.715
127.010
125.636
117.950
117.380
116.430
74.245
37.651
37.046
24.671
22.741
^{13}C NMR (126 MHz, DMSO-D6) δ 159.62, 158.31, 152.62, 145.13, 142.63, 139.69, 128.94, 127.71, 127.01, 125.64, 117.95, 117.38, 116.43, 74.24, 37.65, 37.05, 24.67, 22.74.
160
150
140
130
120
110
100
90
80
70
60
50
40
30
20
10
0
f1 (ppm)

Figure S10: Potential-time (min.) curves for the corrosion of mild steel in 1.0M H_2SO_4 solution before and after adding concentrations of (E11-13) He1-Ph-Cl, (E21-23) He2-Ph-CH_3, (E31-33) He4-CN and (E41-43) He3-Ph, respectively.

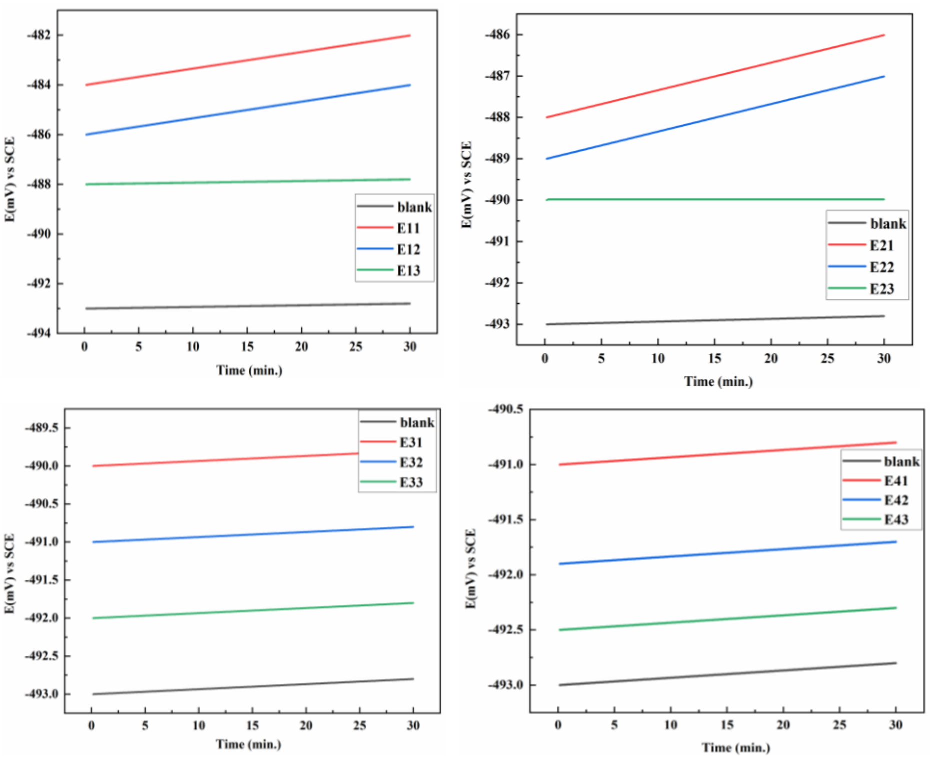

Figure S11: Mulliken charge population analysis MCPA for all tested inhibitors.

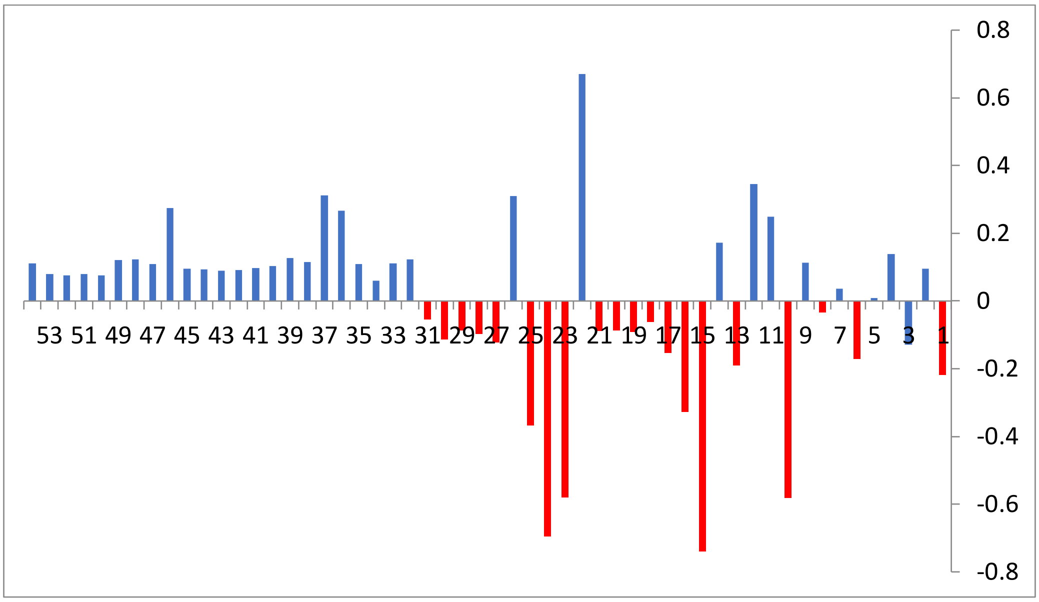

Table S1. Values of emission CIE coordinates of 7,8-dihydrothienoisoquinolines

Wavelength excitation	He1-Ph-Cl (x, y)	He2-Ph-CH_3 (x, y)	He3-Ph (x, y)	He4-CN (x, y)
$\lambda_{ex.}$ 315 nm	0.267, 0.488	0.306, 0.540	0.283, 0.530	0.146, 0.463
$\lambda_{ex.}$ 360 nm	0.267, 0.488	0.300, 0.534	0.265, 0.475	0.251, 0.467
$\lambda_{ex.}$ 390 nm	0.279, 0.515	0.305, 0.541	0.131, 0.521	0.254, 0.475
$\lambda_{ex.}$ 450 nm	0.288, 0.532	0.296, 0.543	0.283, 0.530	0.254, 0.412
Emitted color	Green	Yellowish green	Green	Green

Table S2. Potential (mV) against time (min.) of mild steel exposed to 1.0M H_2SO_4 with different types of DHTQ derivatives

Inhibitors (ppm)		-Eim	-Es.s
1.0M H_2SO_4		493	494
He1-Ph-Cl	100	488	488
	200	486	486
	500	480	484
He2-Ph-CH_3	100	490	490
	200	487	489
	500	486	488
He4- CN	100	492	492
	200	491	491
	500	490	490
He3-Ph	100	492	493
	200	492	492
	500	491	491

Table S3: Thermodynamic parameters using Langmuir adsorption isotherm of mild steel surface in 1.0 M H_2SO_4 containing different concentrations of the DHTQ derivatives.

Test solution	$LogK_{ads}$	Slope	R^2	- ΔG_{ads} (kj/mol)
He1-Ph-Cl	1.792	1.027	0.999	20.2
He2-Ph-CH_3	1.699	1.100	0.999	19.6
He4- CN	1.859	1.173	0.999	20.6
He3-Ph	1.698	1.200	0.999	19.6
